# α-Synuclein accumulation and GBA deficiency due to L444P GBA mutation contributes to MPTP-induced parkinsonism

**DOI:** 10.1186/s13024-017-0233-5

**Published:** 2018-01-08

**Authors:** Seung Pil Yun, Donghoon Kim, Sangjune Kim, SangMin Kim, Senthilkumar S. Karuppagounder, Seung-Hwan Kwon, Saebom Lee, Tae-In Kam, Suhyun Lee, Sangwoo Ham, Jae Hong Park, Valina L. Dawson, Ted M. Dawson, Yunjong Lee, Han Seok Ko

**Affiliations:** 1 Neuroregeneration and Stem Cell Programs, Institute for Cell Engineering, The Johns Hopkins University School of Medicine, Baltimore, MD USA; 2 Department of Neurology, Baltimore, MD USA; 3 Department of Physiology, Baltimore, MD USA; 4 Solomon H. Snyder Department of Neuroscience, Baltimore, MD USA; 5 Department of Pharmacology and Molecular Sciences, The Johns Hopkins University School of Medicine, Baltimore, MD USA; 6 Division of Pharmacology, Department of Molecular Cell Biology, Sungkyunkwan University School of Medicine, Samsung Biomedical Research Institute, Suwon, South Korea; 7 Samsung Medical Center (SMC), Sungkyunkwan University School of Medicine, Samsung Biomedical Research Institute, Suwon, South Korea; 8 Adrienne Helis Malvin Medical Research Foundation, New Orleans, LA USA; 9 Diana Helis Henry Medical Research Foundation, New Orleans, LA USA

**Keywords:** Parkinson’s disease, GBA, MPTP, Mitochondrial dysfunction, α-synuclein

## Abstract

**Background:**

Mutations in glucocerebrosidase (GBA) cause Gaucher disease (GD) and increase the risk of developing Parkinson’s disease (PD) and Dementia with Lewy Bodies (DLB). Since both genetic and environmental factors contribute to the pathogenesis of sporadic PD, we investigated the susceptibility of nigrostriatal dopamine (DA) neurons in L444P GBA heterozygous knock-in (GBA^*+/L444P*^) mice to 1-methyl-4-phenyl-1,2,3,6-tetrahydropyridine (MPTP), a selective dopaminergic mitochondrial neurotoxin.

**Method:**

We used GBA^*+/L444P*^ mice, α-synuclein knockout (SNCA^*−/−*^) mice at 8 months of age, and adeno-associated virus (AAV)-human GBA overexpression to investigate the rescue effect of DA neuronal loss and susceptibility by MPTP. Mitochondrial morphology and functional assay were used to identify mitochondrial defects in GBA^*+/L444P*^ mice. Motor behavioral test, immunohistochemistry, and HPLC were performed to measure dopaminergic degeneration by MPTP and investigate the relationship between GBA mutation and α-synuclein. Mitochondrial immunostaining, qPCR, and Western blot were also used to study the effects of α-synuclein knockout or GBA overexpression on MPTP-induced mitochondrial defects and susceptibility.

**Results:**

L444P GBA heterozygous mutation reduced GBA protein levels, enzymatic activity and a concomitant accumulation of α-synuclein in the midbrain of GBA^*+/L444P*^ mice. Furthermore, the deficiency resulted in defects in mitochondria of cortical neurons cultured from GBA^*+/L444P*^ mice. Notably, treatment with MPTP resulted in a significant loss of dopaminergic neurons and striatal dopaminergic fibers in GBA^*+/L444P*^ mice compared to wild type (WT) mice. Levels of striatal DA and its metabolites were more depleted in the striatum of GBA^*+/L444P*^ mice. Behavioral deficits, neuroinflammation, and mitochondrial defects were more exacerbated in GBA^*+/L444P*^ mice after MPTP treatment. Importantly, MPTP induced PD-like symptoms were significantly improved by knockout of α-synuclein or augmentation of GBA via AAV5-hGBA injection in both WT and GBA^*+/L444P*^ mice. Intriguingly, the degree of reduction in MPTP induced PD-like symptoms in GBA^*+/L444P*^α-synuclein (SNCA)^*−/−*^ mice was nearly equal to that in SNCA^*−/−*^ mice after MPTP treatment.

**Conclusion:**

Our results suggest that GBA deficiency due to L444P GBA heterozygous mutation and the accompanying accumulation of α-synuclein render DA neurons more susceptible to MPTP intoxication. Thus, GBA and α-synuclein play dual physiological roles in the survival of DA neurons in response to the mitochondrial dopaminergic neurotoxin, MPTP.

**Electronic supplementary material:**

The online version of this article (10.1186/s13024-017-0233-5) contains supplementary material, which is available to authorized users.

## Background

Parkinson’s disease (PD) is a multi-factorial neurodegenerative disorder characterized by the loss of DA neurons and motoric dysfunction [1]. Non-motor symptoms of PD include cognitive and mental problems, autonomic failure, and sleep disorders [2]. PD is also neuropathologically characterized by the accumulation of pathological α-synuclein, which is a major component of Lewy bodies (LBs) and Lewy neurites (LNs) [3].

Gaucher’s disease is the most common lysosomal storage disorder that is caused by an inherited enzymatic deficiency of glucocerebrosidase (GBA) that cleaves the glucose moiety from glucocerebroside [4]. Subjects with GBA mutations have higher risk for developing PD, increased accumulation of pathological LBs, and more cognitive changes than those without GBA mutations [5]. PD patients with GBA mutations have an earlier onset of PD and exhibit more cognitive deficits than subjects without GBA mutations and are generally less responsive to L-3,4-dihydroxyphenylalanine (L-Dopa) [6, 7]. PD patients are five times more likely to have GBA mutations than unaffected patients [8]. In addition, people with Gaucher mutations are more likely to have family members with PD [9]. N370S and L444P mutations in GBA are common mutations in GBA-associated PD [10]. Moreover, patients with dementia and Lewy Body (DLB) have a higher frequency of GBA mutations [11]. Still, Gaucher carriers and most patients with Gaucher’s disease do not develop parkinsonism, suggesting that although Gaucher mutations contribute to vulnerability to parkinsonism in some patients, other genetic and/or environmental factors might be involved in the phenotype [6].

It is thought that sporadic PD might be caused by a combination of both genetic susceptibilities and environmental factors [1]. Although the discovery of PD-causing genes has provided new insights into pathogenesis of familial PD, little information is available about the etiology of the most common sporadic form of PD. Mutations in GBA represent a risk or susceptibility factor for developing sporadic PD [12]. However, research trials have provided little evidence that a GBA deficiency could interact with environmental factors to increase the risk of sporadic PD. Therefore, it is important to understand how mutations in GBA and related factors such as α-synuclein affect the survival of dopaminergic neurons due to environmental factors in sporadic PD. The MPTP animal model of PD recapitulating PD-like symptoms is a valuable tool that has been used to understand the molecular mechanisms underlying dopaminergic neurodegeneration in sporadic PD [13–16]. Interestingly, GBA inhibition promotes MPTP/MPP^+^ toxicity in experimental Parkinson disease [17]. The aim of the present study was to understand the role of GBA deficiency due to the L444P GBA mutation in dopaminergic neurodegeneration and how this genetic risk factor of GBA deficiency might interact with the environmental toxin, MPTP. To address this, L444P GBA heterozygous knock-in (GBA^*+/L444P*^) mice at 8 months of age and age-matched control littermates were evaluated after MPTP intoxication. Furthermore, GBA^*+/L444P*^ mice were cross-bred with α-synuclein knock-out (SNCA^*−/−*^) mice or subjected to adeno-associated virus (AAV)-GBA stereotaxic injection. All resulting animals were subjected to MPTP intoxication. Using these animals, the dopaminergic system was assessed via unbiased stereological counting and quantitative assessment combined with immunohistochemistry using tyrosine hydroxylase (TH) antibody and glial fibrillary acidic protein (GFAP), high-performance liquid chromatography (HPLC), behavioral analysis, and biochemical studies. Our results showed that haplodeficiency due to L444P GBA mutation could lead to mitochondrial defects in primary neurons. Moreover, we showed that vulnerability to MPTP-induced DA neurons loss, striatal fiber loss, DA depletion, motor deficits, mitochondrial defects, and glial activation were exacerbated in GBA^*+/L444P*^ mice. These defects were rescued by deletion of α-synuclein and overexpression of GBA in GBA^*+/L444P*^ mice. Taken together, our results suggest that physiological role of GBA and its related α-synuclein is required to protect DA neurons against MPTP mitochondrial neurotoxins.

## Methods

### Animals

All experimental procedures were in accordance with the guidelines of Laboratory Animal Manual of National Institute of Health Guide for the Care and Use of Animals. They were approved by the Johns Hopkins Medical Institute Animal Care and Use Committee. GBA^*+/L444P*^ mice were generated by Dr. Richard L. Proia [18] and obtained from the Mutant Mouse Regional Resource Center (MMRRC) at University of North Carolina (B6; 129S4-*Gba*^*tm1Rlp*^/MMRRC Stock#: 000117-UNC). α-Synuclein KO (SNCA^*−/−*^, B6;129X1-*Snca* ^*tm1Rosl/J*^) mice were obtained from Jackson Laboratory (ME, USA). To prepare genomic DNA isolation for genotyping, mouse tail was incubated with 200 μl of DirectPCR Lysis reagent (Viagen biotech, CA, USA) containing 0.5 mg/ml of Proteinase K (Sigma-Aldrich, MO, USA) at 55 °C for 24 h followed by incubation at 85 °C for 45 min. Then, 1 μl of the lysate was added to PCR reaction buffer containing 10 mM dNTPs, 20 μM forward and reverse primers, 1 μl of AmpliTaq® DNA polymerase, and 1 X GeneAmp® PCR buffer II (Applied Biosystems, CA, USA). PCR reaction was performed using Veriti thermal cycler (Applied Biosystems). PCR conditions for GBA^*+/L444P*^ mice were as follows: 94 °C for 10 min, 36 cycles of 94 °C for 45 s, 62 °C for 45 s, and 72 °C for 60 s, followed by 72 °C for 10 min. Primer sequences used for PCR were as follows: forward, 5’-CCC CAG ATG ACT TGA TGC TGG-3′; reverse, 5’-CCA GGT CAG GAT CTC TGA TGG-3′. PCR product size was approximately 200 bp. PCR product was digested with restriction endonuclease NciI (New England BioLabs, MA, USA) for 2 h at 37 °C. Enzyme digested PCR products were subjected to 2% agarose gel electrophoresis to determine genotypes of mice. PCR conditions for SNCA^*−/−*^ mice were as follows: 94 °C for 2 min as the first step; 10 cycles of 95 °C for 20 s, 65 °C for 15 s, and 68 °C for 10 s as the second step; 28 cycles of 94 °C for 15 s, 60 °C for 15 s, 72 °C for 10 s as the third step followed by 72 °C for 1 min. Primer sequences used for the PCR were as follows: wild type forward, 5’-GGC GAC GTG AAG GAG CCA GGG A -3′; wild type reverse, 5’-CAG CGA AAG GAA AGC CGA GTG ATG TAC T-3′; mutant forward, 5’-CTG AAT GAA CTG CAG GAC GA-3′; mutant reverse, 5′-ATA CTT TCT CGG CAG GAG CA-3′. PCR product size for the wild type was approximately 320 bp. For mutant, PCR product size was approximately 172 bp. PCR products were subjected to 2% agarose gel electrophoresis to determine genotypes of mice.

### MPTP intoxication in mice

**GBA**^***+/L444P***^ **mice groups**: (1) fifteen WT mice with saline, (2) fifteen WT mice with MPTP, (3) fifteen GBA^*+/L444P*^ mice with saline, (4) fifteen GBA^*+/L444P*^ mice with MPTP.

**GBA**^***+/L444P***^**SNCA**^***−/−***^ **mice groups**: (1) six WT mice with saline, (2) six GBA^*+/L444P*^ mice with saline, (3) seven SNCA^*−/−*^ mice with saline, (4) seven GBA^*+/L444P*^SNCA^*−/−*^ mice with saline, (5) seven WT mice with MPTP, (6) six GBA^*+/L444P*^ mice with MPTP, (7) six SNCA^*−/−*^ mice with MPTP, (8) seven GBA^*+/L444P*^SNCA^*−/−*^ mice with MPTP.

**GBA**^***+/L444P***^ **with AAV-GBA mice groups**: (1) six AAV-GFP stereotaxic injected WT mice with saline, (2) eight AAV-GBA stereotaxic injected WT mice with saline, (3) seven AAV-GFP stereotaxic injected GBA^*+/L444P*^ mice with saline, (4) six AAV-GBA stereotaxic injected GBA^*+/L444P*^ mice with saline, (5) six AAV-GFP stereotaxic injected WT mice with MPTP, (6) seven AAV-GBA stereotaxic injected WT mice with MPTP, (7) seven AAV-GFP stereotaxic injected GBA^*+/L444P*^ mice with MPTP, (8) six AAV-GBA stereotaxic injected GBA^*+/L444P*^ mice with MPTP. Mice at 8 months of age received four intraperitoneal (IP) injections of MPTP-HCl (20 mg/kg free base, Sigma-Aldrich, MO, USA) or saline control at 2 h intervals [19]. All mice were sacrificed at 7 days after the last MPTP injection and brain samples were processed for biochemical and immunohistochemistry studies.

### Generation of adeno-associated virus (AAV) vectors

To overexpress human GBA (hGBA) in vivo, an AAV5-hGBA was generated using a gene construct composed of human GBA cDNA (Genbank RefSeq: BC003356.1) and eGFP cDNA by Vector Biolabs (PA, USA). The expression of hGBA and eGFP was driven by the same CAG2 promoter and linked with a T2A peptide. These constructs in pAAV vector were then packaged in AAV serotype 5 through transfections using HEK293 cells. Viral particles were purified and concentrated by serial CsCl_2_ centrifugation (Vector Biolabs, PA, USA). Expression of GBA was confirmed by immunoblotting and immunostaining using mouse primary cultured neuron. AAV5 virus with empty vector expressing EGFP only was used as control.

### Stereotaxic intranigral injection of virus

For stereotaxic injection of AAV5-GFP and AAV5-hGBA, 8-month-old mice of indicated genotypes were anesthetized with pentobarbital (60 mg/kg). An injection cannula (26.5 gauge) was stereotaxically applied to the substantia nigra pars compacta (SNpc), unilaterally into the right hemisphere (anteroposterior, 3.2 mm from bregma; mediolateral, 1.3 mm; dorsoventral, 4.3 mm). Infusion was performed at a rate of 0.4 μl per min. High titer AAV5-GFP and AAV5-hGBA (5.0 ~ 3.5 × 10^13^ VG per ml in PBS) were injected into each mouse. After the final injection, the injection cannula was maintained in the SNpc for an additional of 5 min for complete absorption of virus. It was then slowly removed from the mouse brain and the scalp was closed by suturing. Wound healing and recovery were monitored following surgery. After AAV-virus stereotaxic injection for 1 month, mice received four intraperitoneal (IP) injections of MPTP-HCl (20 mg/kg free base, Sigma-Aldrich, MO, USA) or saline control at 2 h intervals [19]. For biochemical experiments and stereological analysis, all mice were sacrificed at 7 days after the last MPTP injection. Specifically, for stereological analysis, animals were perfused with ice-cold PBS and fixed intracardially with 4% paraformaldehyde.

### Monoamine analysis

High performance liquid chromatography with electrochemical detection (HPLC-ECD) was performed to measure biogenic anime concentrations as described previously [20]. Briefly, mice were euthanized by decapitation and striatum samples were collected on ice. Striatal tissues were weighed and sonicated in 0.2 ml ice cold 0.01 mM perchloric acid containing 0.01% EDTA and 60 ng of 3,4-dihydroxybenzylamine (DHBA) as an internal standard followed by centrifugation (15,000×g, 30 min, 4 °C). The supernatant was passed through a 0.2 mm filter and 20 μl of the supernatant was injected to the HPLC column (4.6 mm × 150 mm C-18 reverse phase column, MC Medical, Tokyo, Japan) using a dual channel coulochem III electrochemical detector (Model 5300, ESA Inc., Chelmsford, MA, USA). Protein concentrations of tissue homogenates were measured using BCA protein assay kit (Pierce, Rockford, IL, USA). Data were normalized to protein concentration and expressed in ng/mg protein.

### Immunohistochemistry and quantification

Mice were perfused with ice-cold phosphate buffered saline (PBS) and then fixed with 4% paraformaldehyde/PBS (pH 7.4). Brains were collected and post-fixed for 4 h in PBS containing 4% paraformaldehyde and incubated in PBS solution containing 30% sucrose (pH 7.4). Brains were then frozen in OCT compound and serial coronal sections were cut in thickness of 30 μm using a microtome. Free-floating 30 μm sections were blocked with 4% goat serum/PBS with 0.2% Triton X-100 and incubated with an antibody against TH (rabbit polyclonal; Novus Biologicals, Littleton, CO, USA), followed by incubation with biotin-conjugated anti-rabbit secondary antibody (anti-rabbit polyclonal; Vector Labs). After triple washing steps, ABC reagents (Vector Labs) were added and sections were developed using SigmaFast DAB Peroxidase Substrate (Sigma-Aldrich). Sections were counterstained with Nissl (0.09% thionin).

TH-positive and Nissl positive DA neurons from the SNpc region were counted through optical fractionators, an unbiased method for cell counting. This method was carried out using a computer-assisted image analysis system consisting of an Axiophot photomicroscope (Carl Zeiss Vision, Jena, Germany) equipped with a computer-controlled motorized stage (Ludl Electronics, NY, USA), a Hitachi HV C20 camera, and Stereo Investigator software (MicroBright-Field, MBF Bioscience, VT, USA). The total number of TH-stained neurons and Nissl counts was analyzed as described previously [20]. Fiber density in the striatum was analyzed by optical density (OD) measurement. ImageJ software (NIH, http://rsb.info.nih.gov/ij/) was used to analyze OD as described previously [20].

Astrocytes of the SNpc region were stained with anti-GFAP (1:2000; Agilent-Dako, CA, USA) antibody followed by incubation with biotin-conjugated anti-rabbit antibody and ABC reagents. Sections were developed using SigmaFast DAB Peroxidase Substrate (Sigma-Aldrich). Densities of astrocytes in the SNpc region were measured with ImageJ software.

### Immunofluorescence analysis

Immunofluorescence was performed on 30 μm thick serial brain sections. For immunofluorescence studies, paraformaldehyde (4%)/PBS (pH 7.4)-fixed coronal brain sections were blocked with 10% donkey serum (Jackson Immunoresearch)/PBS plus 0.3% Triton X-100 and incubated in antibodies to TH (Novus Biologicals), SDHA (Cell signaling), GFAP (Agilent-Dako), and Tuj1(Biolegned), for overnight at 4 °C. After brief washes with PBS, floating brain sections were incubated in 0.1% Triton X-100 and 5% donkey serum in PBS, followed by 1 h of incubation in a mixture of FITC-conjugated (Jackson Immunoresearch), CY3-conjugated (Jackson Immunoresearch), and Alexa-Fluor 647-conjugated (Jackson Immunoresearch) secondary antibodies at room temperature. The fluorescent images were acquired via a Zeiss confocal microscope (Zeiss Confocal LSM 710) after the coverslips were mounted with DAPI mounting solution (VECTASHIELD HardSet Antifade Mounting Medium with DAPI, Vector laboratories). All images were processed by the Zeiss Zen software. The selected area in the signal intensity range of the threshold was measured using ImageJ analysis.

### Immunoblot analysis

Mouse brain tissues or mouse neurons were homogenized in lysis buffer (10 mM Tris-HCL, pH 7.4, 150 mM NaCl, 5 mM EDTA, 0.5% Nonidet P-40, 10 mM Na-β-glycerophosphate) containing phosphate inhibitor mixture I and II (Sigma-Aldrich) and complete protease inhibitor mixture (Roche, IN, USA) using a Diax 900 homogenizer (Sigma-Aldrich). After homogenization, samples were rotated at 4 °C for 30 min to obtain complete lysis. These lysates were centrifuged at 22000×g for 20 min to collect supernatants. Protein concentrations were measured using BCA Kit (Pierce, IL, USA) with BSA as standard. Proteins were then subjected to immunoblot analyses. Briefly, equal amounts of protein (10-20 μg) prepared from mouse ventral midbrain (VMB) were resolved on 8-16% or 4-20% gradient SDS-PAGE gels and then transferred to nitrocellulose membranes. These membranes were blocked with blocking solution (Tris-buffered saline with 5% non-fat dry milk and 0.1% Tween-20) for 1 h and incubated in anti-TH (1:2000; Novus Biologicals), anti-GBA (1: 1000, Sigma-Aldrich), anti-α-synuclein (1: 2000; BD Biosciences, CA, USA), anti-α-synuclein oligomers (1: 1000, Agrisera, Vännäs, SWEDEN), anti-α-synuclein filament (1:1000, Abcam, Cambridge, UK), anti-succinate dehydrogenase complex subunit A (SDHA, 1:1000, Cell signaling, MA, USA), anti-pyruvate dehydrogenase (PDH, 1:1000, Cell signaling), anti-voltage-dependent anion channels (VDAC, 1:1000, Cell signaling), anti-SQSTM1/p62 (1:1000, Abcam), or anti-LC3A/B-I/II (1:1000, Cell signaling) antibodies at 4 °C overnight followed by incubation with HRP-conjugated rabbit of mouse secondary antibodies (1: 50,000; GE Healthcare) and HRP-conjugated mouse of donkey secondary antibodies (1: 10,000; GE Healthcare) for 1 h at room temperature (RT). Immunoblot signals were visualized by enhanced chemiluminescence (Thermo Scientific, IL, USA). These membranes were subsequently used for re-probing with HRP-conjugated β-actin antibody (1:50,000; Sigma-Aldrich).

### Filter trap dot blot assay

For dot blot analysis, TX-soluble and TX-insoluble lysates were diluted in 1% SDS-PBS and boiled for 5 min. Immediately after cooling, the indicated amount of proteins were loaded onto nitrocellulose membranes (0.2 μm pore size) settled on a dot blotter (Bio-Rad).

### Primary neuronal cultures

Primary neurons were prepared from wild-type (WT; GBA^*+/+*^) or GBA^*+/L444P*^ mice at embryonic day 15 as described previously [21]. Dissociated cells were plated onto poly-*D*-lysine coated dishes with culture medium (Neurobasal Media, Gibco, CA, USA) containing B27 supplement and L-Glutamine (Gibco). These cultures were maintained in 7% CO_2_ incubator at 37°C. Culture media were refreshed twice a week. To inhibit glial cell growth, 30 μM 5-fluoro-2′-deoxyuridine was added to cultures at 5 days after culture.

### Mitochondrial morphology assessment

Primary neurons were plated onto glass coverslips coated with poly-*D*-lysine at density of 10,000 cells/cm^2^. After 10 days of in vitro (DIV) culture, neurons were stained with MitoTracker® Orange CMTMRos probes (Life technologies, OR, USA) following manufacturer’s instructions. Mitochondria were then imaged using a Zeiss confocal microscope (Zeiss Confocal LSM 710). Mitochondrial morphological characteristics such as length and aspect ratio (AR, the ratio between major and minor axis of the ellipse equivalent to the mitochondrion) were quantified using ImageJ software.

### Reactive oxygen species (ROS) measurements

Primary neurons were plated onto the 6 well plates coated with poly-*D*-lysine at density of 500,000 cells/well. After 10 DIV, ROS levels were measured using 5-(and-6)-chloromethyl-2′,7′-dichlorodihydrofluorescein diacetate acetyl ester (CM-H2DCFDA, C6827, Life technologies) following the manufacturer’s instructions. Briefly, primary neurons were removed from growth media and centrifuged at 300×g for 5 min. These cells were then incubated in PBS containing 1 μM of CM-H2DCFDA for 30 min at 37°C. Their fluorescence intensities were measured on a Perkin Elmer plate reader (excitation wavelength, 492-495 nm; emission wavelength, 517-527 nm).

### Activity of mitochondrial complex I enzyme

Activity of mitochondrial complex I enzyme was measured using Complex I Enzyme Activity Kit (Abcam) according to the manufacturer’s protocol. Briefly, primary cortical neurons were plated onto poly-*D*-lysine coated 6 cm dishes at a density of 1,000,000 cells/dish. At 10 DIV, proteins were extracted with 1/10 volume of detergent containing PBS. Samples were then centrifuged at 12000×g for 20 min. Then, 100 μg of proteins was loaded onto complex I pre-coated microplate and incubated at RT for 3 h. After the incubation, the plate was rinsed twice with washing buffer followed by the addition of 200 μl of assay solution. Complex I enzyme activity was then determined after measuring OD at wavelength 450 nm (OD450) at approximate interval of 1 min for 30 min.

### Determination of oxygen consumption rate

Primary cortical neurons prepared from WT and GBA^*+/L444P*^ mice were plated onto the poly-*D*-lysine coated Seahorse 24 well culture plate at a density of 500,000 cells/well. At 10 DIV, neurons were washed with 37°C PBS and incubated in Seahorse assay medium at 37°C for 1 h. The plate was then loaded in an XF96 analyzer to measure oxygen consumption rate (OCR). Oligomycin, carbonyl cyanide m-chlorophenyllhydrazone (CCCP), and rotenone were used sequentially to access basal respiration, coupling of respiratory chain, and mitochondrial respiratory capacity, respectively. OCR was measured with a protocol of 1 min of mix, 1 min of wait, and 2 min of measurement at 37°C. It was normalized relative to protein concentration in each well.

### Transmission electron microscopy (TEM) imaging

To obtain TEM images, WT and GBA^*+/L444P*^ mice were deeply anesthetized with pentobarbital (60 mg/kg) and perfused with PBS containing 1% sodium nitrite (pH 7.4) followed by fixation in a solution containing 3% paraformaldehyde, 1.5% glutaraldehyde, 0.1 M cacodylate, and 2.5% sucrose (pH 7.4). After 5 min of continuous perfusion, brains were harvested and post-fixed for 1 h. After washing with 0.1 M cacodylate and 2.5% sucrose buffer (pH 7.4), brains were post-fixed with Palade’s 1% OsO_4_ solution [22]. After post-fixation, brains were rinsed with Kellenberger solution and distilled water. After dehydration with series of cold ethanol, brains were embedded and TEM images were collected using a Philips EM 410 TEM equipped with a Soft Imaging System Megaview III digital camera (Olympus, Tokyo, Japan). Mitochondrial length and aspect ratio (AR, the ratio between the major and the minor axis of the ellipse equivalent to the mitochondrion) were quantified using ImageJ software.

### Determination of mitochondria DNA (mtDNA) copy number using real-time quantitative PCR

Total DNA was isolated from ventral midbrain tissues using TRIzol reagent (Life technologies) following the manufacturer’s instruction. Real-time quantitative PCR was performed to determine the relative quantity of mtDNA using ViiA™ 7 real-time PCR system and SYBR GreenER reagent as described previously [23]. The following primers were used: *GAPDH* forward: 5′-TGG GTG GAG TGT CCT TTA TCC-3′, *GAPDH* reverse: 5′-TAT GCC CGA GGA CAA TAA GG-3′; mtDNA *COX1* forward: 5’-GCC TTT CAG GAA TAC CAC GA-3′, mtDNA *COX1* reverse: 5′-AGG TTG GTT CCT CGA ATG TG-3′; mtDNA *CYTB* forward: 5′-ATT CCT TCA TGT CGG ACG AG-3′; and mtDNA *CYTB* reverse: 5’-ACT GAG AAG GCC CCC TCA AAT-3′.

### Glucocerebrosidase (GCase) activity assay

GCase activity assay was performed as described previously [24, 25]. Briefly, mouse ventral midbrain tissues were homogenized in a buffer containing 0.25 M sucrose, 10 mM HEPES (pH 7.4), and 0.1 M EDTA followed by centrifugation (6800×g at 4 °C for 5 min). The resulting supernatant was further centrifuged at 17,000×g for 10 min and the pellet enriched with lysosomes was collected in 50 μl of activity assay buffer (0.25% Triton X-100 (Sigma-Aldrich), 0.25% Taurocholic acid (Sigma-Aldrich), and 1 mM EDTA in citrate/phosphate buffer, pH 5.4). GCase activity was measured by adding 50 μl of 1% BSA, 1 mM 4-Methylumbelliferyl β-glucophyranoside (4-MU; Sigma-Aldrich), and/or 10 mM conduritol B epoxide (CBE, Sigma-Aldrich). After incubation at 37 °C for 40 min, 50 μl (equal-volume) of 1 M glycine (pH 12.5) was added to terminate the reaction. Sample volume was 100 μl per well in 96-well plate (Thermo Fisher). Fluorescence was measured using a Perkin Elmer plate reader (excitation wavelength, 355 nm; emission wavelength, 460 nm, 0.1 s). GCase activity was obtained by subtracting GCase activity in presence of CBE from total GCase activity of each sample. CBE treatment resulted in 95-97% of reduction in GCase activity. Absolute enzyme activity was determined based on fluorescence intensity using 4-MU standard curve generated for human recombinant GCase (R&D systems, MN, USA).

### 20S proteasome activity assay

Primary neurons were plated onto 6 cm dishes coated with poly-D-lysine at density of 1,000,000 cells/dish. After 10 DIV, 20S proteasome activity was measured using 20S Proteasome assay kit (Cayman chemical, #10008041) following the manufacturer’s instructions. Briefly, primary neurons were removed from growth media and centrifuged at 500×g for 5 min. The cells were then incubated in 200 μl of the 20S proteasome assay buffer and 100 μl of the 20S proteasome lysis buffer for 30 min at RT. And then, cells were centrifuged 1000×g for 10 min and 90 μl of supernatant were transferred with 10 μl of the substrate solution to a black 96-well assay plate. The Fluorescent intensity of each well was measured using microplate reader (Molecular Devices, SpectraMax Gemini XS, excitation = 360 nm; emission = 480 nm).

### Measurement of lysosomal calcium concentration

SH-SY5Y cells were transfected with constructs for 48 h by Lipofectamine (Thermo Fisher Scientific) following the manufacturer’s instructions. After 48 h, transfected SH-SY5Y cells were plated onto glass coverslips coated with poly-*D*-lysine at density of 10,000 cells/cm^2^. SH-SY5Y cells were loaded with Oregon Green 488 BAPTA-1 dextran (Invitrogen, 100 μg/ml) for 24 h. For staining of lysosome, CellLight lysosomes (LAMP-1)-RFP (Invitrogen) was also loaded to the SH-SY5Y cells. In vitro calcium-binding (*K*_*d*_) affinities of Oregon Green 488 BAPTA-1 were determined using calcium calibration buffer kit (Invitrogen) adjusted to pH 4.5. The calibration curve was obtained as described previously [26]. In vitro minimal and maximal fluorescence (F_min_ and F_max_) were calculated by perfusing the ionomycin, nigercin, and valinomycin pre-treated SH-SY5Y cells with 0 or 10 mM Ca^2+^ external solutions. The equation: [Ca^2+^]_ly_ = *Kd* × (F-F_min_)/(F_max_-F) were used for calculation.

### Behavioral tests

#### Pole test

Animals were acclimatized in a behavioral procedure room for 30 min. The pole was made with 75 cm of metal rod with a diameter of 9 mm. It was wrapped with bandage gauze [20, 27, 28]. Mice were placed on the top of the pole (7.5 cm from the top of the pole) facing the head-up. Total time taken to reach the base of the pole was recorded. Before actual test, mice were trained for two consecutive days. Each training session consisted of three test trials. On the test day, mice were evaluated in three sessions and total time was recorded. The maximum cutoff time to stop the test and recording was 60 s. Results for turn down, climb down, and total time (in sec) were recorded.

#### Grip strength test

Grip strength test was performed using a Bioseb grip strength test machine (BIO-GS3, Bioseb, FL USA) [29]. Performance of mice was assessed three times. To assess grip strength, each mouse was allowed to hold a metal grid with forelimbs. Mouse was lifted by the tail so that its hindlimbs were not in contact with the grid. Mice were gently pulled backwards by the tail until they could no longer hold the grid. Grip strength was scored as grams (g) unit.

### Statistics

Data were presented as mean ± standard deviation of the mean (SEM) with at least 3 independent experiments. Representative morphological images were taken out of at least 3 experiments with similar results. Unpaired two-tailed Student’s test or analysis of two-way ANOVA was performed followed by Bonferroni post hoc analysis to assess statistical significance. A *p* value of less than 0.05 was considered statistically significant.

## Results

### L444P GBA heterozygous mutation reduces GBA levels and activity that is accompanied by accumulation of α-synuclein

**Fig. 1:**
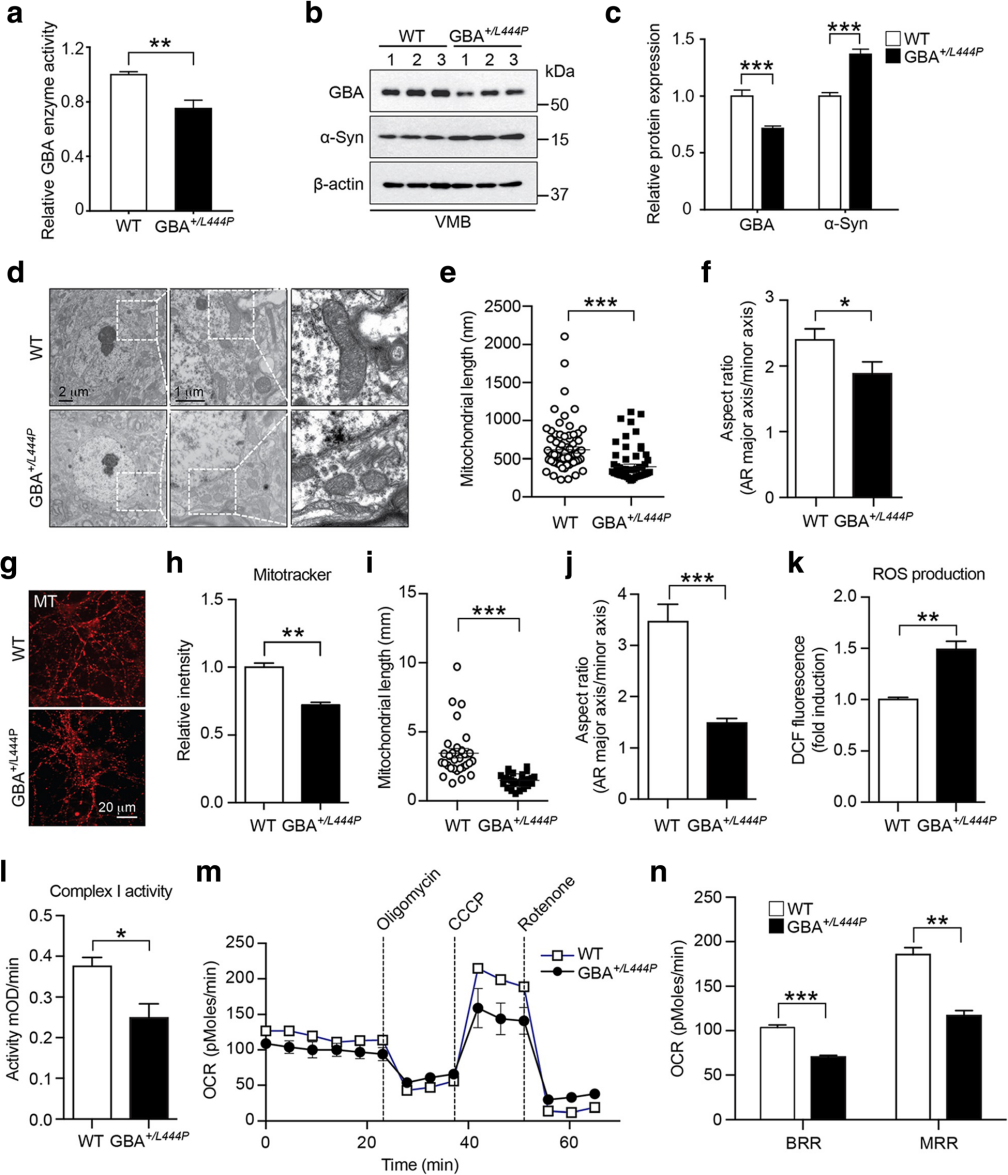
L444P GBA heterozygous mutation leads to GBA abnormalities, accumulation of α-synuclein, and mitochondrial defects both in vitro and in vivo. **a** GBA enzymatic activities were measured using lysosome-enriched fraction samples of ventral midbrain (VMB) in WT or GBA^*+/L444P*^ mice. GBA enzyme activity was normalized against GBA enzyme activity of WT mice. **b** VMB lysates from WT and GBA^*+/L444P*^ were immunoblotted with anti-GBA and α-synuclein antibodies. c GBA and α-synuclein expression levels were normalized against β-actin. **a**, **c** Error bars represent the mean ± S.E.M (n = six mice per group). **d** Representative transmission electron microscopy (TEM) images of mitochondria in the SNpc of WT and GBA^*+/L444P*^ mice. **e** Mitochondrial length and **f** aspect ratio (mitochondrial major axis over minor axis) were measured from littermate WT control (sixty-four mitochondria from seven cells) and GBA^*+/L444P*^ mice (fifty mitochondria from seven cells) group and represented as graph. **g** Representative images of MitoTracker positive structure in WT and GBA^*+/L444P*^ primary cortical neurons (10 DIV). **h** The intensity of MitoTracker positive structure of WT and GBA^*+/L444P*^ primary cultured neurons (n = three mice per group). **i** Mitochondrial length and **j** aspect ratio (mitochondrial major axis over minor axis) are shown (thirty mitochondria from three different images of each group). **k** Reactive oxygen species (ROS) levels were measured in primary cortical neurons of WT and GBA^*+/L444P*^ using CM-H2DCFDA. **l** Mitochondrial complex I enzyme activity in WT and GBA^*+/L444P*^ primary cortical neurons. **m**, **n** Oxygen consumption rate (OCR) was determined by Seahorse assay in WT and GBA^*+/L444P*^ primary cortical neurons. **k**-**n** Error bars represent the mean ± S.E.M (n = six mice per group). Student’s t-test or *P < 0.05, **P < 0.01, ***P < 0.001 vs. WT group

To explore the effect of the L444P GBA heterozygous mutation on brain GBA enzyme activity and protein levels, ventral midbrain (VMB) tissues were collected from GBA^*+/L444P*^ mice at 8 months of age. GBA activity was measured via an enzyme activity assay while GBA protein levels were measured via Western blot analysis. Consistent with the previous study [30], results of the GBA enzyme activity (Fig. 1a) and protein levels (Fig. 1b and c) showed a 30% reduction of GBA in the VMB of GBA^*+/L444P*^ mice compared to that in age-matched WT mice. Since GBA deficiency due to GBA mutations can result in an accumulation of α-synuclein [5, 31, 32], we explored whether the haplodeficiency of GBA due to the L444P GBA heterozygous mutation affects α-synuclein accumulation. To address this, VMB tissues were collected from GBA^*+/L444P*^ mice and tissue lysates were subjected to Western blot analysis using an α-synuclein antibody. Results showed that α-synuclein protein levels were increased by 38.6% in the VMB of GBA^*+/L444P*^ mice compared to those in age-matched WT mice (Fig. 1b and c).

### L444P GBA heterozygous mutation reduces mitochondrial size, decreases mitochondrial complex I activity, and reduces respiration

To determine the effect of L444P GBA heterozygous mutation on mitochondrial morphology, transmission electron microscopic (TEM) images were collected from the VMB of GBA^*+/L444P*^ mice at 8 months of age. Results of the TEM image analysis of mitochondrial size are shown in Fig. 1d. Mitochondria of GBA^*+/L444P*^ mice were smaller in size compared to those of WT mice (Fig. 1d). Quantification of mitochondrial size in the VMB of GBA^*+/L444P*^ mice revealed a 42.9% reduction compared to that of WT mice (Fig. 1d-f). The wellness and size of mitochondria in primary cortical neurons cultured from GBA^*+/L444P*^ mice were also assessed using MitoTracker Orange CMTMRos. Primary cortical neurons carrying L444P GBA heterozygous mutation exhibited a 28.2% reduction in MitoTracker accumulated in active mitochondria (Fig. 1g and h). Mitochondrial size was assessed by measuring the length and aspect ratio (AR) of MitoTracker Orange within each cell as determined using ImageJ software. Mitochondrial size was reduced by 45.4% in primary cortical neurons cultured from GBA^*+/L444P*^ mice compared to that of neurons cultured from WT mice (Fig. 1i and j). To further address whether haplodeficiency of GBA due to the L444P GBA heterozygous mutation affects mitochondrial function, reactive oxygen species (ROS) generation was investigated using a DCF-DA cellular ROS detection assay. Mitochondrial complex I enzyme activity was also assessed from primary cortical neurons cultured from WT and GBA^*+/L444P*^ mice. ROS generation was increased (Fig. 1k), but mitochondrial complex I enzyme activity was decreased (Fig. 1l) in primary cortical neurons harboring the L444P GBA heterozygous mutation compared to those in control neurons (Fig. 1k and l). Furthermore, the oxygen consumption rate (OCR) of primary cortical neurons was measured using a XF-24 analyzer (Seahorse Bioscience). Primary cortical neurons carrying L444P GBA heterozygous exhibited a 67.8% reduction in basal respiration (Fig. 1m) and 63.1% reduction in carbonyl cyanide m-chlorophenyl hydrazine (CCCP)-induced maximal respiration (Fig. 1n). Taken together, these results indicate that expression of L444P GBA heterozygous mutation can lead to mitochondria defects both in vitro and in vivo. In addition to mitochondrial dysfunction, we observed reduction of proteasome activity by GBA deficiency (Additional file 1: Figure S1).

### Enhanced susceptibility of GBA^*+/L444P*^ mice to MPTP-induced PD-like symptoms

**Fig. 2:**
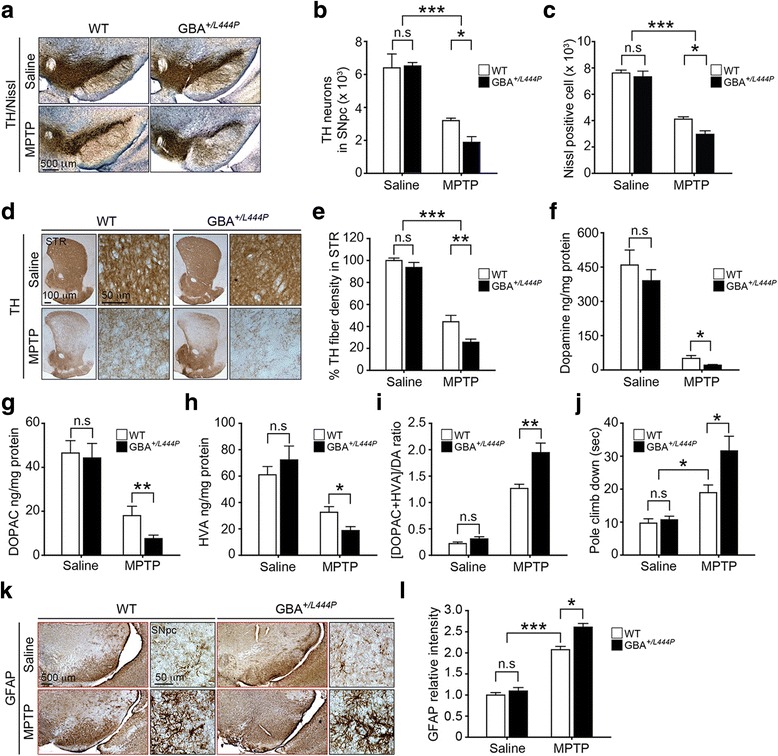
Effect of L444P GBA heterozygous mutation on the susceptibility of mice to MPTP-induced PD-like symptoms. **a** Representative photomicrographs from coronal mesencephalon sections containing TH-positive neurons in WT and GBA^*+/L444P*^ mice treated with saline or MPTP, respectively (scale bar, 500 μm). **b** Stereology counts of TH and **c** Nissl-positive neurons in the SNpc region. Unbiased stereologic counting was performed for the SNpc region.) Representative photomicrograph of striatal sections stained for TH immunoreactivity with low (scale bar, 100 μm) and high magnification (scale bar, 50 μm). **e** Quantification of dopaminergic fiber densities in the striatum using Image J software (NIH). **a**-**e** Error bars represent the mean ± S.E.M (n = ten mice per group). Striatal DA and metabolites levels were measured by HPLC-ECD. Levels of **f** DA, **g** DOPAC, and **h** HVA in the striatum from WT and GBA^*+/L444P*^ mice treated with saline or MPTP were measured. **i** DA turnover [(DOPAC + HVA/DA)] in the striatum was calculated. **f**-**i** Error bars represent the mean ± S.E.M (n = four mice per group). **j** Pole test was conducted on the sixth day post MPTP injection. Maximum time to climb down the pole was limited to 60 s. Error bars represent the mean ± S.E.M (n = fifteen mice per group). **k** Representative images of immunohistochemistry data for glial fibrillary acidic protein (GFAP, astrocyte specific marker) with low (scale bar, 100 μm) and high magnification (scale bar, 50 μm). **l** Intensities of GFAP positive signals in the SNpc of WT and GBA^*+/L444P*^ mice treated with saline or MPTP were quantified and shown as a graph. **k**, **l** Error bars represent the mean ± S.E.M (ten mice per group). Two-way ANOVA was used for statistical analysis followed by *post-hoc* Bonferroni test for multiple group comparison. *P < 0.05, **P < 0.01, ***P < 0.001 vs. MPTP-treated WT group, or saline-treated WT and GBA^*+/L444P*^ group. n.s: not significant

To examine whether the L444P GBA heterozygous mutation could enhance the susceptibility of mice to loss of DA neurons following MPTP administration (Additional file 2: Figure S2), the number of TH-positive neurons in the SNpc was assessed via an unbiased stereological counting analysis [33]. Representative TH immunostained images of the SNpc sections (Fig. 2a) and quantification of the number of TH- and Nissl-positive stained DA neurons (Fig. 2b and c) revealed a significant loss of DA neurons in both WT and GBA^*+/L444P*^ mice treated with MPTP compared to mice treated with saline. Importantly, the L444P GBA heterozygous mutation significantly increased the loss of DA neurons in response to MPTP compared to that of WT mice treated with MPTP (Fig. 2b and c).

To assess the effect of the L444P GBA heterozygous mutation on striatal TH-immuno-positive fiber density, TH immunostained images were collected from striatal sections. These TH stained images were analyzed by optical densitometry using Image J software (NIH) [34]. MPTP treatment significantly reduced the striatal dopaminergic fiber density in both WT control and GBA^*+/L444P*^ mice compared to saline-treatment (Fig. 2d and e). Notably, there was a greater reduction in TH-fiber density in GBA^*+/L444P*^ mice after MPTP treatment compared to that in WT mice treated with MPTP (Fig. 2d and e).

To determine the effect of the L444P GBA heterozygous mutation on DA metabolism, levels of DA and its metabolites in the striatum were measured by reverse-phase HPLC-electrochemical detection (ECD) for all genotypes. MPTP treatment significantly reduced DA levels in both WT and GBA^*+/L444P*^ mice (Fig. 2f). Importantly, there was a greater reduction in DA levels in GBA^*+/L444P*^ mice with MPTP treatment compared to that in WT mice treated with MPTP (Fig. 2f). MPTP treatment also significantly reduced levels of 3,4-dihydroxyphenylacetic acid (DOPAC) (Fig. 2g) and homovanillic acid (HVA) (Fig. 2h) in the striatum of WT and GBA^*+/L444P*^ mice compared to those treated with saline. Notably, there was a greater reduction in the levels of DOPAC and HVA in GBA^*+/L444P*^ mice with MPTP treatment compared to those in WT mice treated with MPTP (Fig. 2g and h). To determine whether there was a catabolic alteration of DA in GBA^*+/L444P*^ mice, DA turnover ratios in the striatum was calculated from all genotypes. The DA turnover ratio was significantly increased in both WT and GBA^*+/L444P*^ mice with MPTP treatment (Fig. 2i). Importantly, there was a greater increase in the DA turnover ratio in GBA^*+/L444P*^ mice with MPTP treatment compared to WT mice (Fig. 2i).

To examine the role of the L444P GBA heterozygous mutation on behavioral deficits induced by MPTP administration, motor deficits were assessed by the pole test. MPTP treatment significantly increased the time to reach the base of the pole for both WT and GBA^*+/L444P*^ mice compared to that for saline-treated control mice (Fig. 2j). Notably, there was a greater increase in the time to reach the base of the pole in GBA^*+/L444P*^ mice with MPTP treatment compared to that of WT mice treated with MPTP (Fig. 2j).

To assess the effect of the L444P GBA heterozygous mutation on astrocyte activation, glial fibrillary acidic protein (GFAP) staining was conducted within the SN regions of all genotypes. GFAP-stained images were analyzed by Image J software [35, 36]. MPTP treatment significantly enhanced astrocyte activation in both WT and GBA^*+/L444P*^ mice compared to saline-treatment (Fig. 2k and l). Importantly, there was a greater increase in the GFAP immunoreactivity in the SN of GBA^*+/L444P*^ mice with MPTP treatment compared to that in MPTP treated WT mice (Fig. 2k and l).

On the other hand, L444P GBA heterozygous mutation alone in the absence of MPTP treatment had no effect on the number of DA neurons (Fig. 2a-c), striatal fiber density (Fig. 2d and e), levels of DA and its metabolites, DA turnover ratio (Fig. 2f-i), behavior (Fig. 2j), or gliosis (Fig. 2k and l) of GBA^*+/L444P*^ mice at 8 months of age.

### Depletion of α-synuclein prevents the susceptibility of GBA^*+/L444P*^ mice to MPTP-induced PD-like symptoms

**Fig. 3:**
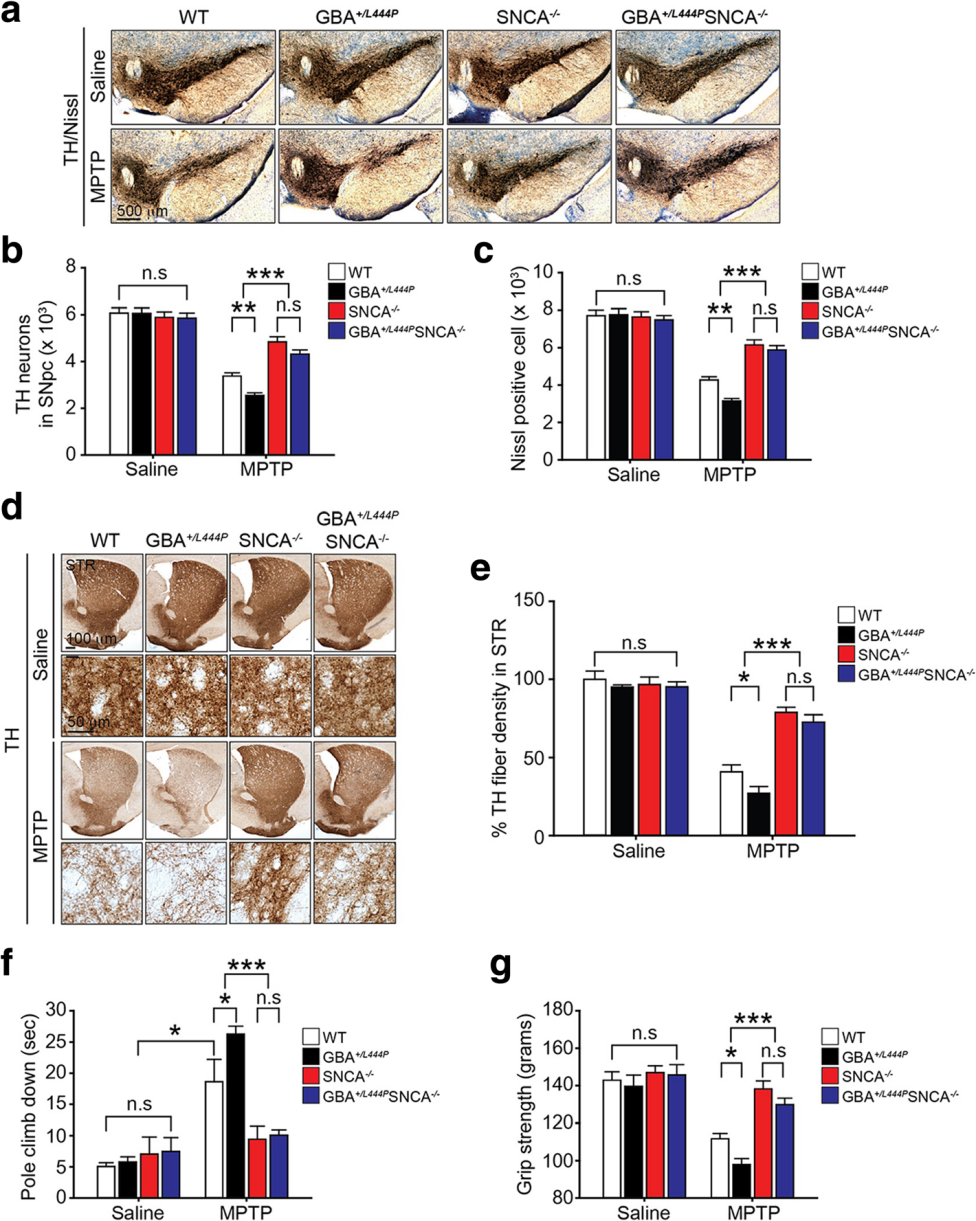
Effect of α-synuclein on susceptibility of GBA^*+/L444P*^ mice to MPTP-induced PD-like symptoms. **a** Representative photomicrographs from coronal mesencephalon sections containing TH-positive neurons in littermate WT control, GBA^*+/L444P*^, SNCA^*−/−*^, and SNCA^*−/-*^GBA^*+/L444P*^ mice treated with saline or MPTP, respectively (scale bar, 500 μm). **b** Stereology counts of TH, and **c** Nissl-positive neurons in the SNpc region. Unbiased stereologic counting was performed for the SNpc region. **d** Representative photomicrograph of striatal sections stained for TH immunoreactivity with low (scale bar, 100 μm) and high magnification (scale bar, 50 μm). **e** Quantification of dopaminergic fiber densities in the striatum using Image J software (NIH). **a**-**e** Error bars represent the mean ± S.E.M (n = four mice per group). **f** Pole test was conducted on the sixth day post MPTP injection. **g** Grip strength test was conducted on the sixth day post MPTP injection. Behavioral abnormalities and susceptibility in pole test and grip strength test induced by MPTP injection were ameliorated in SNCA^*−/−*^ and SNCA^*−/-*^GBA^*+/L444P*^ mice. Maximum time to climb down the pole was limited to 60 s. **f**, **g** Error bars represent means ± S.E.M (n = six or seven mice per group). Two-way ANOVA was used for statistical analysis followed by *post-hoc* Bonferroni test for multiple group comparison. *P < 0.05, **P < 0.01, ***P < 0.001 vs. MPTP-treated WT group, or MPTP-treated WT and GBA^*+/L444P*^ group. n.s: not significant

Our results showed that α-synuclein levels were increased in GBA^*+/L444P*^ mice (Fig. 1b and c), consistent with results of a previous study [37]. Accordingly, we sought to investigate whether α-synuclein might play a key role in the susceptibility to MPTP-induced PD-like symptoms in mice harboring the L444P GBA heterozygous mutation by crossing GBA^*+/L444P*^ mice with α-synulcein knock-out (SNCA^*−/−*^) mice. The resulting mice were subjected to MPTP intoxication and their loss of DA neurons was assessed using TH immunohistochemistry-based stereologic cell counting. DA neurons in SNCA^*−/−*^ mice were found to be significantly resistant to MPTP intoxication (Fig. 3a-c), consistent with results of prior studies [38, 39]. Importantly, the enhanced MPTP induced loss of DA neurons in the GBA^*+/L444P*^ mice was significantly protected by depletion of α-synuclein (Fig. 3a-c). Next, we assessed the role of α-synuclein on the MPTP induced reduction of striatal dopaminergic fiber density. SNCA^*−/−*^ and GBA^*+/L444P*^SNCA^*−/−*^ mice failed to show a reduction in striatal TH-immuno-positive fiber density induced by MPTP (Fig. 3d and e). We also investigated the effect of depletion of α-synuclein on the behavioral deficits induced by MPTP. The motor deficits induced by MPTP were significantly decreased in both SNCA^*−/−*^ mice and GBA^*+/L444P*^SNCA^*−/−*^ mice as assessed by the pole test and grip strength (Fig. 3f and g). Astrocyte activation in response to MPTP was assessed by immunohistochemistry using GFAP. The astrocyte activation induced by MPTP was significantly decreased in both SNCA^*−/−*^ mice and the GBA^*+/L444P*^SNCA^*−/−*^ mice (Additional file 3: Figure S3a and b). The degree of restoration of MPTP induced DA neurodegeneration (Fig. 3a-c), loss of striatal dopaminergic fiber density (Fig. 3d and e), motor deficits (Fig. 3f and g), and astrocyte activation (Additional file 3: Figure S3a and b) in the GBA^*+/L444P*^SNCA^*−/−*^ mice was similar to those of SNCA^*−/−*^ mice treated with MPTP.

### α-Synuclein depletion restores the decreased mitochondria markers following MPTP intoxication of GBA^*+/L444P*^ mice

**Fig. 4:**
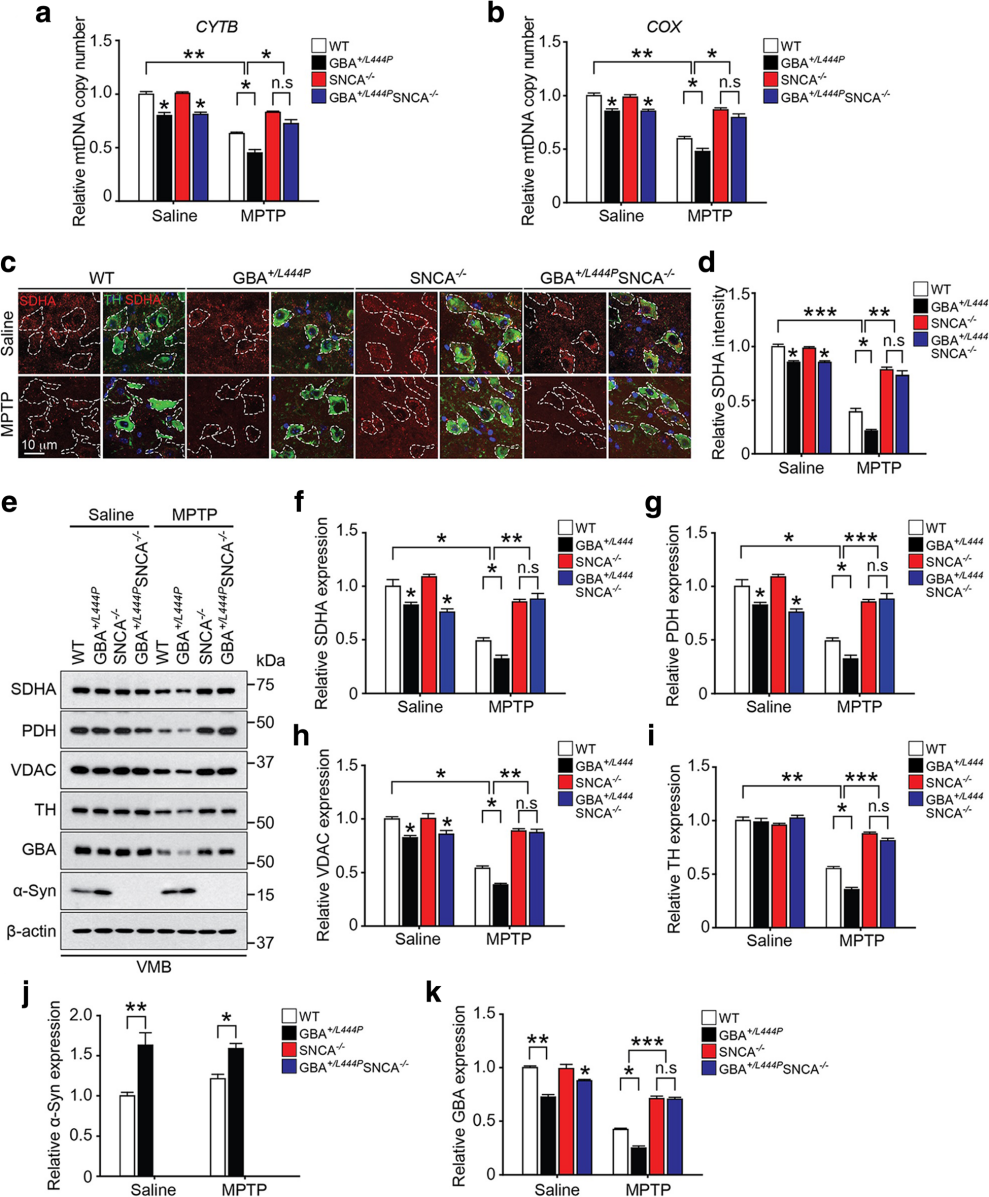
Deficiency of α-synuclein leads to decreased susceptibility of GBA^*+/L444P*^ mice to MPTP-induced mitochondrial defects. **a**, **b** Relative quantity of mitochondrial DNA (mtDNA) in the ventral midbrain was measured using two different mtDNA markers (*CYTB* and *COX*) normalized to GAPDH. **c** Representative immunofluorescent images of TH (green), SDHA (red), and DAPI (blue). White dot line is shown in TH neurons. **d** Intensities of SDHA positive signals in the SNpc of mice treated with saline or MPTP were quantified and shown as a graph. **e** Immunoblots of SDHA, PDH, VDAC, TH, α-synuclein, and GBA. VMB lysates were immunoblotted with anti-SDHA, anti-PDH, anti-VDAC, anti-TH, anti-α-synuclein, and anti-GBA antibodies. **f** SDHA, **g** PDH, **h** VDAC, **i** TH, **j** α-synuclein, and **k** GBA expression levels were normalized against β-actin. **a**-**k** Error bars represent the mean ± S.E.M (n = three mice per group). Two-way ANOVA was used for statistical analysis followed by *post-hoc* Bonferroni test for multiple group comparison. *P < 0.05, **P < 0.01, ***P < 0.001 vs. saline-treated WT or saline-treated GBA^*+/L444P*^ or MPTP-treated WT group. n.s: not significant

Since the L444P GBA heterozygous mutation decreased the number of mitochondria in neurons and tissues (Fig. 1d-j), we investigated the effect of depletion of α-synuclein on the sensitivity of mitochondria markers in response to MPTP in GBA^*+/L444P*^ mice. Mitochondrial DNA (mtDNA) copy number was assessed by measuring two different mitochondrial markers, cytochrome b (CYTB) and cytochrome c oxidase (COX), by real-time quantitative PCR using the VMB of WT, GBA^*+/L444P*^, SNCA^*−/−*^, and GBA^*+/L444P*^SNCA^*−/−*^ mice with or without MPTP intoxication. WT and GBA^*+/L444P*^ mice treated with MPTP showed a significant reduction in CYTB and COX copy number (Fig. 4a and b). Notably, there was a greater reduction in CYTB and COX copy number in GBA^*+/L444P*^ mice treated with MPTP compared to that in WT mice treated with MPTP (Fig. 4a and b). The reduction in mitochondrial DNA copy number in both WT and GBA^*+/L444P*^ mice treated with MPTP was significantly restored by depletion of α-synuclein (Fig. 4a and b). Further analyses revealed that MPTP treatment reduced protein levels of mitochondrial succinate dehydrogenase complex flavoprotein subunit A (SDHA) in the VMB of both WT and GBA^*+/L444P*^ based on immunofluorescence (Fig. 4c and d) and Western blot analyses (Fig. 4e and f). The reduction of SDHA protein level was greater in GBA^*+/L444P*^ mice treated with MPTP compared to that in WT mice treated with MPTP. Importantly, the reduction of SDHA protein level in both WT and GBA^*+/L444P*^ mice treated with MPTP was significantly restored by depletion of α-synuclein (Fig. 4c-f). Similar results were observed for mitochondrial protein levels of pyruvate dehydrogenase complex (PDH) (Fig. 4e and g), voltage-dependent anion channels (VDAC) (Fig. 4e and h), and TH protein levels (Fig. 4e and i) as assessed by Western blot analysis. Intriguingly, GBA protein levels in both WT and GBA^*+/L444P*^ mice treated with MPTP were decreased. The reduction in GBA level was greater in GBA^*+/L444P*^ mice treated with MPTP compared to that in WT mice treated with MPTP. The reduction in GBA protein level was recovered in both WT and GBA^*+/L444P*^ mice by depletion of α-synuclein (Fig. 4e and k). Depletion of α-synuclein was confirmed in all genotypes of mice with or without MPTP treatment (Fig. 4e and j). Intriguingly, there was no difference in the levels of α-synuclein monomers between GBA^*+/L444P*^ mice treated with saline and GBA^*+/L444P*^ mice treated with MPTP (Fig. 4j). However, the levels of α-synuclein oligomers were three times higher in the GBA^*+/L444P*^ mice treated with MPTP than in the WT mice treated with MPTP (Additional file 4: Figure S4a). There was no α-synuclein fibril formation in both WT and GBA^*+/L444P*^ mice treated with MPTP as assessed by dot blot analysis (Additional file 4: Figure S4b). Our data suggests that MPTP contributes to the conversion of α-synuclein monomers to oligomers and the conversion could be exacerbated by GBA deficiency due to L444P GBA mutation in vivo.

The degree of restoration of the MPTP-induced reduction of mitochondrial DNA copy number (Fig. 4a and b), mitochondrial proteins (Fig. 4c-h), and the reduction in TH (Fig. 4i) and GBA proteins (Fig. 4k) in GBA^*+/L444P*^SNCA^*−/−*^ mice were similar to those in SNCA^*−/−*^ mice treated with MPTP. On the other hand, intriguingly, we found that overexpression of wild type α-synuclein in SH-SY5Y cells reduced lysosomal calcium levels while augmentation of GBA in the cells overexpressing wild type α-synuclein rescued the defect as measured by ratiometric methods via confocal microscopy (Additional file 5: Figure S5).

### Augmentation of GBA ameliorates the susceptibility of GBA^*+/L444P*^ mice to MPTP-induced PD-like symptoms

**Fig. 5:**
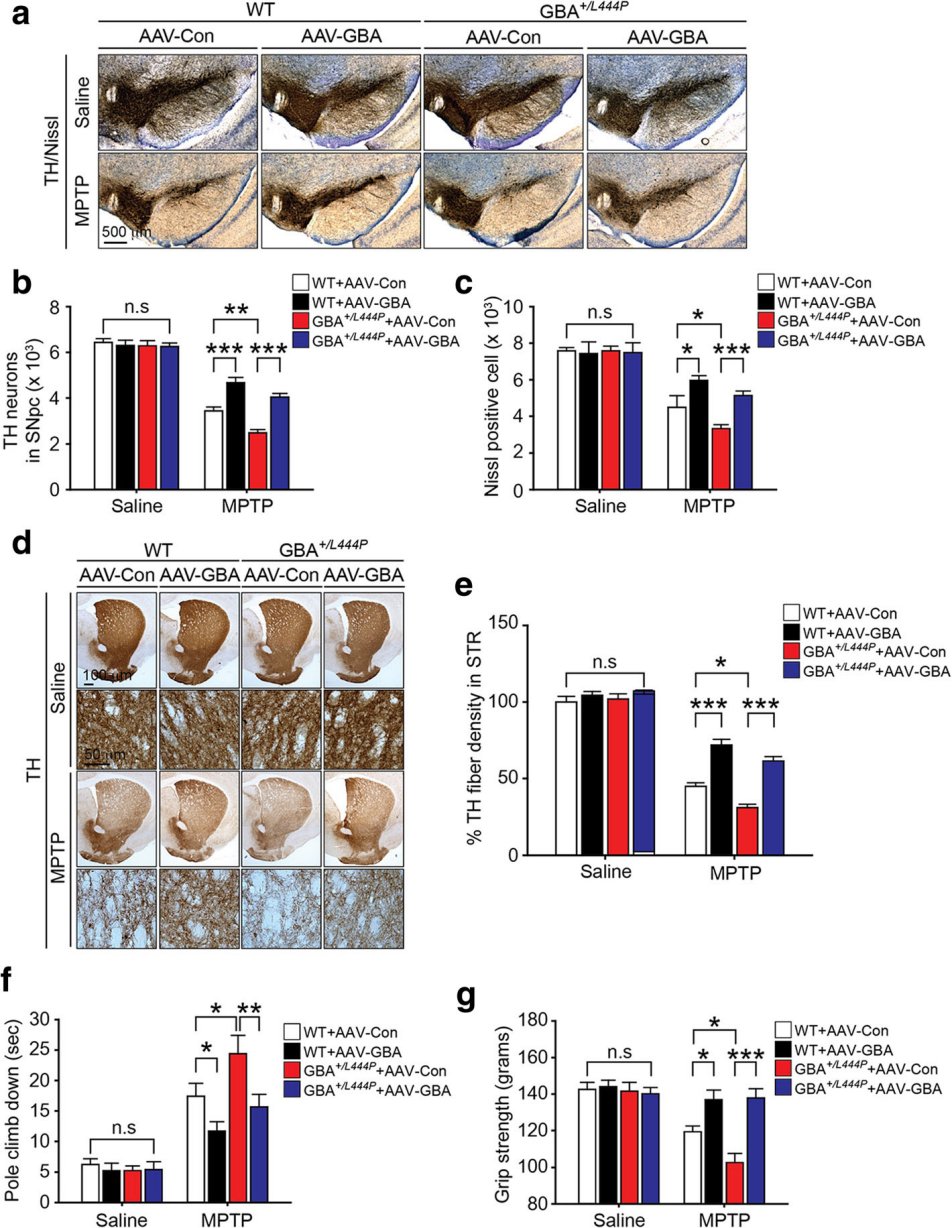
Effect of GBA overexpression on susceptibility of GBA^+/L444P^ mice to MPTP-induced PD-like symptoms. **a** Representative photomicrographs from coronal mesencephalon sections containing TH-positive neurons in AAV-Con injected WT, AAV-GBA injected WT, AAV-Con injected GBA^*+/L444P*^, and AAV-GBA injected GBA^*+/L444P*^ mice treated with saline or MPTP, respectively (scale bar, 500 μm). **b** Stereology counts of TH, and **c** Nissl-positive neurons in the SNpc region. Unbiased stereologic counting was performed in the SNpc region. **d** Representative photomicrograph of striatal sections stained for TH immunoreactivity with low (scale bar, 100 μm) and high magnification (scale bar, 50 μm). **e** Quantification of dopaminergic fiber densities in the striatum using Image J software (NIH). **a**-**e** Error bars represent means ± S.E.M (n = five mice per group). **f** Pole test was conducted on the sixth day post MPTP injection. **g** Grip strength test was conducted on the sixth day post MPTP injection. Behavioral abnormalities and susceptibility in pole test and grip strength test induced by MPTP injection were ameliorated in WT and GBA^*+/L444P*^ mice with AAV-GBA. **f**, **g** Error bars represent the mean ± S.E.M (six or seven mice per group for behavioral studies). Two-way ANOVA was used for statistical analysis followed by *post-hoc* Bonferroni test for multiple group comparison. *P < 0.05, **P < 0.01, ***P < 0.001 vs. MPTP-treated WT group with AAV-Con or MPTP-treated GBA^*+/L444P*^ group with AAV-Con. n.s: not significant

Many studies have suggested that augmentation of GBA activity has beneficial effect on GBA mutation-associated neurodegeneration [37]. Our results revealed that GBA levels and activity were decreased in GBA^*+/L444P*^ mice (Fig. 1a-c). Therefore, we sought to investigate whether augmentation of GBA via adeno-associated virus (AAV) transduction could reduce the susceptibility of GBA^*+/L444P*^ to MPTP. WT and GBA^*+/L444P*^ mice with AAV5-GFP (control) or AAV5-hGBA injection were subjected to MPTP intoxication and DA neuron number was assessed via TH immunohistochemistry-based unbiased stereological counting (Additional file 6: Figure S6). GBA overexpression was achieved with AAV5-hGBA constructed with the CAG2 promoter along with GFP fluorescence reporter co-expression (Additional file 7: Figure S7a and b). Overexpression of GBA in the SNpc in both WT and GBA^*+/L444P*^ mice led to a significant protection against MPTP-induced loss of DA neurons (Fig. 5a-c). To assess the effect of GBA augmentation on striatal TH-immuno-positive fiber density, TH staining images were collected from striatal sections and analyzed. Overexpression of GBA via AAV5-hGBA injection restored the reduction in TH-fiber density induced by MPTP in both WT and GBA^*+/L444P*^ mice (Fig. 5d and e). To examine the effect of GBA augmentation on behavioral deficits induced by MPTP administration, motor deficits were assessed by the pole test (Fig. 5f) and grip strength (Fig. 5g). Behavioral defects induced by MPTP were significantly restored by overexpression of GBA in both WT and GBA^*+/L444P*^ mice (Fig. 5f and g). To assess the effect of GBA augmentation on astrocyte activation, GFAP staining images collected from SN regions of all genotypes of mice were analyzed. Overexpression of GBA in the SNpc of both WT mice and GBA^*+/L444P*^ mice significantly reduced MPTP-induced astrocyte activation (Additional file 3: Figure S3c and d).

Next, the effect of GBA augmentation on the levels of mitochondrial proteins were assessed by Western blot analyses. Reduced levels of mitochondrial SDHA (Additional file 8: Figure S8a and b), PDH (Additional file 8: Figure S8a and c), VDAC (Additional file 8: Figure S8a and d) mitochondrial proteins, TH (Additional file 8: Figure S8a and e), and α-synuclein (Additional file 8: Figure S8a and g) induced by MPTP were significantly restored by overexpressing GBA in the SNpc of both WT and GBA^*+/L444P*^ mice. Overexpression of GBA was confirmed in all genotypes of mice with or without MPTP treatment (Additional file 8: Figure S8a and f).

## Discussion

Our results showed that GBA deficiency, a genetic risk factor for PD, interacts with the environmental neurotoxin, MPTP, potentiating the neurotoxic effects of MPTP. In particular, the L444P GBA heterozygous mutation rendered the nigrostriatal DA system more susceptible to MPTP, a potent inhibitor of mitochondrial complex I [40]. The L444P GBA heterozygous mutation resulted in a greater reduction in the loss of nigrostriatal DA neurons, depletion of striatal DA, motor deficits, mitochondrial defects, and glial activation compared to WT mice after MPTP treatment. Importantly, the MPTP-induced PD-like features were reduced by overexpressing GBA or depleting α-synuclein in both WT and L444P GBA heterozygous mice. How the L444P GBA heterozygous mutation increases the susceptibility of mice to MPTP and how overexpression of GBA and α-synuclein null background reduces the susceptibility of mice to MPTP remain unknown. However, it may relate to the levels of α-synuclein, since α-synuclein is required for DA neurotoxicity of MPTP [39].

Mitochondrial dysfunction has been shown to play a crucial role in the pathogeneses of PD [41–43]. In GD animal models, mitochondrial dysfunction, including reduction of mitochondrial membrane potential, reduction of basal and maximal oxygen consumption, and a decrease in reversal of ATPase have been observed [44]. Consistent with these findings, we find accumulation of damaged and fragmented mitochondria as well as defects in complex I activity, respiration, and ROS production in GBA^*+/L444P*^ mice (Fig. 1). These defects in mitochondrial function are likely to account for the GBA^*+/L444P*^ mice being particularly susceptible to MPTP-induced neurotoxicity. Consistent with this notion is our observation that GBA overexpression reduces the MPTP induced defects in both GBA^*+/L444P*^ and WT mice (Fig. 5). The accumulation of damaged and fragmented mitochondria may be due to a defect in lysosomes due to the lysosomal GBA deficiency [44]. Since emerging evidences suggest that damaged and fragmented mitochondria can be removed through mitophagy initiated by the accumulation of PINK1 that triggers recruitment of Parkin to damaged mitochondria [45, 46], it will be important in future studies to explore the relationship of PINK1/Parkin mitophagy pathway and reductions in the level GBA activity.

It has been suggested that loss of GBA function may contribute to neurodegeneration in GBA-associated PD through both a loss- and gain-of-function [47]. α-synuclein is a protein that accumulates and aggregates in PD and drives the neurodegeneration in PD [1, 3, 48]. It is thought that α-synuclein may, in part, be removed through autophagy-lysosome pathway and there appears to be a reciprocal relationship between α-synuclein and lysosomal GBA activity as well as between autophagy and lysosomal GBA activity [25, 44, 49]. Thus, lysosomal dysfunction and autophagy inhibition due to GBA deficiency could lead to a reduction in α-synuclein degradation and a concomitant increase in α-synuclein levels [10, 49]. Our results that α-synuclein levels accumulate in the midbrain of GBA^*+/L444P*^ mice (Fig. 1) is consistent with previous findings in Gaucher’s disease animal models [50] and support the hypothesis that GBA mutations might disrupt cellular pathways related to lysosomal degradation of α-synuclein and the subsequent accumulation of α-synuclein [51]. Previous studies have shown that α-synuclein knockout mice are resistant to MPTP neurotoxicity [39, 52]. Knockout of α-synuclein prevents the enhanced nigrostriatal DA neurodegeneration in response to MPTP in the GBA^*+/L444P*^SNCA^*−/−*^ mice (Fig. 3), suggesting that the increased α-synuclein in these mice accounts for enhanced MPTP neurotoxicity. Intriguingly, a recent study showed that loss-of-function of GBA resulted in increased levels of α-synuclein via inhibition of autophagy [49]. Consistent with this study, WT and GBA^*+/L444P*^ mice treated with MPTP showed a significant increase in SQSTM1/p62 levels and decrease in LC3 levels. The changes in SQSTM1/p62 levels and LC3 were exacerbated in GBA^*+/L444P*^ mice treated with MPTP compared to that in WT mice treated with MPTP (Additional file 9: Figure S9), suggesting that inhibition of autophagy due to GBA mutation could be a mechanistic basis for the increased susceptibility of PD.

It has been previously reported that there is a reciprocal relation between the expression of α-synuclein and GBA protein level and activity [25, 47]. Increased soluble α-synuclein oligomers can impair the transport of newly synthesized GBA protein to the lysosome [25]. GBA protein levels might be regulated by α-synuclein through endoplasmic reticulum (ER) dependent processes [53, 54] and GBA activity is increased in SNCA^*−/−*^ mice [37]. We show that the reduced GBA protein levels observed in the GBA^*+/L444P*^ mice were restored by knocking out α-synuclein in the GBA^*+/L444P*^ mice (Fig. 4e-k). In addition, the drastically reduced GBA protein levels in both WT and GBA^*+/L444P*^ mice after MPTP intoxication were significantly prevented on the SNCA^*−/−*^ background (Fig. 4e-k). Furthermore, overexpression of GBA restored MPTP-induced loss of DA neurons, striatal fiber degeneration, motor defects, astrocyte activation, and α-synuclein levels (Fig. 5 and Additional files 3 and 8: Figure S3 and Figure S8). Accordingly, it is plausible that the accumulation of α-synuclein due to GBA deficiency accounts for the increased susceptibility of DA neurons in the GBA^*+/L444P*^ mice to MPTP-induced neurotoxicity. The recovery of GBA protein and activity might also be critical for the resistance of GBA^*+/L444P*^SNCA^*−/−*^ mice to MPTP-induced neurotoxicity. Taken together, these results are consistent with the reciprocal relationship between expression of α-synuclein and GBA protein and their activity. Moreover, the relative levels of α-synuclein and GBA are major contributors to MPTP-induced PD like features. As shown above, our results show that reducing α-synuclein expression or overexpressing GBA can prevent the dopaminergic neuronal death by environmental toxin (MPTP). The important point is that our experiments were preceded by GBA overexpression or α-synuclein KO prior to MPTP treatment.

Haplodeficiency of GBA due to L444P GBA heterozygous mutation does not directly cause PD-like symptoms in mice. In humans with haplodeficiency of GBA due to GBA heterozygous mutations, it is likely that the following factors individually or combinatorically contribute to the PD phenotype: other genetics factors, modifier genes, environmental factors, and/or lifestyle. [47]. Consistent with this notion is the observation that haplodeficiencey of GBA clearly exacerbates MPTP induced PD-like features. Mutated GBA and/or the accumulated glucocerebroside might disrupt cellular pathways necessary for autophagy-lysosomal degradation. We show that haplodeficiency due to L444P GBA heterozygous mutation can lead to defects such as accumulation of damaged fragmented mitochondria and α-synuclein accumulation, resulting in failed clearance of damaged mitochondria within cells. Further studies are needed to determine whether GBA deficiency interacts with other environmental toxins and insults including pesticides, fungicides, heavy metals, proteasome inhibitors, as well as viral and bacterial infections and the microbiome.

## Conclusions

Taken together, our results clearly establish that the L444P GBA heterozygous mutation exacerbates the MPTP intoxication model of PD. The GBA^*+/L444P*^ mice showed mitochondrial dysfunction and worse PD-like pathology in response to MPTP. The accumulation of α-synuclein due to GBA deficiency was also associated with mitochondrial dysfunction, autophagy inhibition, and increased susceptibility to MPTP. Furthermore, GBA^*+/L444P*^ mice represent a valuable animal model in which the molecular mechanism underlying how environment factors beyond MPTP might play a role in sporadic PD pathogenesis.

## Additional files

Additional file 1: Figure S1. Activity of 20S proteasome was measured in WT and GBA^*+/L444P*^ primary cultured neurons (n = three per each group). Student’s t-test was used to for statistical analysis. *P < 0.05, ***P < 0.001. (PDF 80 kb)

Additional file 2: Figure S2. The schematic diagram depicts the time schedule of intervention and analyses performed. Numerals represent the days experiments were conducted. On 1th day we injected saline or MPTP (2 h interval, 4 times, 20 mg/kg free base) in 8 months WT, GBA^*+/L444P*^, SNCA^−/−^, GBA^*+/L444P*^SNCA^−/−^ mice. On 6th day, the pole and grip strength were performed. On 7th day, mice were sacrificed for indicated studies. Following are animal numbers used for these studies: behavioral (*n* = 10), neurochemical (*n* = 5), immunohistochemistry (n = 5), and biochemical studies (*n* = 4) per each treatment group. (PDF 110 kb)

Additional file 3: Figure S3. Effect of α-synuclein deficiency and GBA overexpression on susceptibility of GBA^*+/L444P*^ mice to MPTP-induced gliosis. **a**, **c** Representative images of immunohistochemistry data for GFAP with low (scale bar, 500 μm) and high magnification (scale bar, 50 μm). **b**, **d** Intensities of GFAP positive signals in the SNpc of mice treated with saline or MPTP were quantified and shown as a graph. Error bars represent the mean ± S.E.M (n = four mice per group). Two-way ANOVA was used to test for statistical analysis followed by *post-hoc* Bonferroni test for multiple group comparison. *P < 0.05, ***P < 0.001 vs. MPTP-treated WT or GBA^*+/L444P*^ with AAV5-Con or MPTP-treated GBA^*+/L444P*^ with AAV5- Con. n.s: not significant. (PDF 3329 kb)

Additional file 4: Figure S4. MPTP-induced α-synuclein oligomer. **a** Filter trap soluble-α- synuclein oligomer species assay from ventral midbrain of WT and GBA^*+/L444P*^ mice with or without MPTP. Error bars represent the mean ± S.E.M. (n = four mice per group). Two-way ANOVA was used for statistical analysis followed by *post-hoc* Bonferroni test for multiple group comparison. ***P < 0.001 vs. MPTP-treated WT. N.D: not detection. **b** Filter trap insoluble-α-synuclein filament species assay from ventral midbrain of WT and GBA^*+/L444P*^ mice with or without MPTP. α-Synuclein preformed fibril (PFF) is positive control. (PDF 574 kb)

Additional file 5: Figure S5. Lysosomal calcium concentration. SH-SY5Y cells were transfected with indicated constructs for 48 h. The cells were then labeled with CellLight® Lysosome-RFP (LAMP1; red) and loaded with 0.1 mg/ml of lysosomal calcium indicator Oregon Green BAPTA-1 dextran (BAPTA-1; green) for 12 h. The Oregon Green BAPTA-1 signals that co-localized to lysosome (LAMP-1; red) were used for measuring lysosomal calcium concentration. [Ca^2+^]_lys_ was measured using ratiometric methods via confocal microscopy. Two-way ANOVA was used to test for statistical analysis followed by *post-hoc* Bonferroni test for multiple group comparison. ***P < 0.001. (PDF 1122 kb)

Additional file 6: Figure S6. The schematic diagram depicts the time AAV5 injection schedule of intervention and analyses performed. Numerals represent the days’ experiments were conducted. For stereotaxic injection of AAV5-GFP and AAV5-hGBA, 8-month-old mice of indicated genotypes were anesthetized with pentobarbital (60 mg/kg). An injection cannula (26.5 gauge) was stereotaxically applied to the substantia nigra pars compacta (SNpc). After AAV5 stereotaxic injection for 1 month, we injected saline or MPTP (2 h interval, 4 times, 20 mg/kg free base) in WT, and GBA^*+/L444P*^ mice. On 6th day the pole and grip strength test were performed. On 7th day, mice were sacrificed for indicated studies. Following are animal numbers used for these studies: behavioral (*n* = 6-8), neurochemical (n = 4), immunohistochemistry (n = 4), and biochemical studies (n = 4) per each treatment group. (PDF 126 kb)

Additional file 7: Figure S7. Neuron specific AAV5-hGBA overexpression in SNpc region. **a** Vector design of AAV5 hGBA. **b** Representative immunofluorescent images of GFP (green, injection marker), GFAP (red, astrocyte, non-neuronal marker), Tuj1 (Violet, neuronal marker), and DAPI (Blue). (PDF 4671 kb)

Additional file 8: Figure S8. GBA overexpression inhibits MPTP-reduced mitochondrial protein level in GBA^*+/L444P*^ mice. **a** Immunoblots of SDHA, PDH, VDAC, TH, GBA, and α-synuclein from AAV5-Con injected WT, AAV5-hGBA injected WT, AAV5-Con injected heterozygous, and AAV5-hGBA injected heterozygous mice treated with saline or MPTP. VMB lysates were immunoblotted with anti-SDHA, anti-PDH, anti-VDAC, anti-TH, and anti-GBA antibodies. **b** SDHA, **c** PDH, **d** VDAC, **e** TH, **f** GBA, and **g** α-synuclein expression levels were normalized against β-actin. Error bars represent the mean ± S.E.M. (n = three mice per group). Two-way ANOVA was used for statistical analysis followed by *post-hoc* Bonferroni test for multiple group comparison. *P < 0.05, **P < 0.01, ***P < 0.001 vs. saline-treated WT with AAV-Con or saline-treated GBA^*+/L444P*^ with AAV5-Con or MPTP-treated WT with AAV5-Con or MPTP-treated GBA^*+/L444P*^ with AAV5-Con group. (PDF 683 kb)

Additional file 9: Figure S9. L444P GBA heterozygous mutation leads to autophagy abnormality. **a**, **b** Effect of rapamycin on GBA^*+/L444P*^ expression. Primary cortical neurons were cultured from WT and GBA^*+/L444P*^ mice. After 10 DIV, primary neurons were treated with 20 nM of rapamycin (mTOR inhibitor for inducing autophagy) for 24 h. **a** Representative Immunoblots of GBA. **b** GBA expression levels were normalized against β-actin and the error bars represent the mean ± S.E.M (n = four per group). **c** Immunoblots of Autophagy marker proteins SQSTM1/p62, and LC3A/B-I/II. VMB lysates were immunoblotted with anti-SQSTM1/p62, and anti-LC3A/B-I/II antibodies. **d** SQSTM1/p62, and **e** LC3A/B-II expression levels were normalized against β-actin. Error bars represent the mean ± S.E.M (n = four mice per group). Two-way ANOVA was used for statistical analysis followed by *post-hoc* Bonferroni test for multiple group comparison. *P < 0.05, **P < 0.01, ***P < 0.001. n.s: not significant. (PDF 338 kb)

## Abbreviations

4-MU: Methylumbelliferyl β-glucophyranoside
AAV: Adeno-associated virus
AR: Aspect ratio
CBE: Conduritol B epoxide
CCCP: Carbonyl cyanide m-chlorophenyl hydrazine
DA: Dopamine
DHBA: 3,4-dihydroxybenzylamine
DLB: Dementia with Lewy Body
DOPAC: 3,4-dihydroxyphenylacetic acid
ECD: Electrochemical detection
GBA: Glucocerebrosidase
GD: Gaucher’s disease
HPLC: High-performance liquid chromatography
HVA: Homovanillic acid
IP: Intraperitoneal
KI: Knock-in
KO: Knock-out
LBs: Lewy bodies
LNs: Lewy neurites
MPTP: 1-Methyl-4-Phenyl-1,2,3,6-Tetrahydropyridine
mtDNA: Mitochondrial DNA
OCR: Oxygen consumption rate
OD: Optical density
PBS: Phosphate buffered saline
PDH: Pyruvate dehydrogenase
SDHA: Succinate dehydrogenase
SNCA: α-synuclein
SNpc: Substantia nigra pars compacta
TEM: Transmission electron microscopy
TH: Tyrosine hydroxylase
VDAC: Voltage-dependent anion channels
VMB: Ventral midbrain
WT: Wild-type
