# Supplementary material for: α-Synuclein accumulation and GBA deficiency due to L444P GBA mutation contributes to MPTP-induced parkinsonism
Source: Mol Neurodegener. 2018 Jan 8;13:1. doi: 10.1186/s13024-017-0233-5 (PMC5759291; doi:10.1186/s13024-017-0233-5)
Supplement: Supplementary file 1 — Activity of 20S proteasome was measured in WT and GBA+/L444P primary cultured neurons (n = three per each group). Student’s t-test was used to for statistical analysis. *P < 0.05, ***P < 0.001. (PDF 80 kb) [file 13024_2017_233_MOESM1_ESM.pdf]

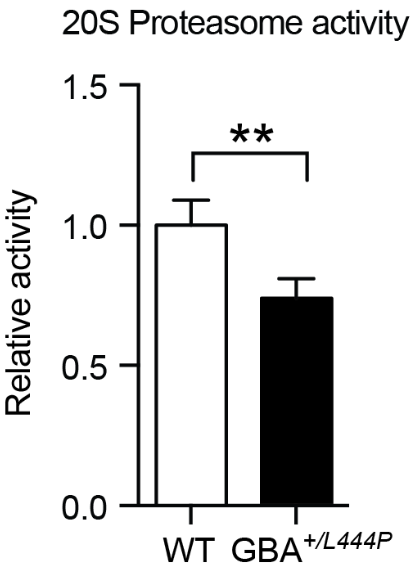

Supplementary Figure 1. Activity of 20S proteasome was measured in WT and GBA$^{+/L444P}$ primary cultured neurons (n= three per each group). Student's t-test was used to for statistical analysis. *P < 0.05, ***P < 0.001.
