# Supplementary material for: α-Synuclein accumulation and GBA deficiency due to L444P GBA mutation contributes to MPTP-induced parkinsonism
Source: Mol Neurodegener. 2018 Jan 8;13:1. doi: 10.1186/s13024-017-0233-5 (PMC5759291; doi:10.1186/s13024-017-0233-5)
Supplement: Supplementary file 2 — The schematic diagram depicts the time schedule of intervention and analyses performed. Numerals represent the days experiments were conducted. On 1th day we injected saline or MPTP (2 h interval, 4 times, 20 mg/kg free base) in 8 months WT, GBA+/L444P, SNCA−/−, GBA+/L444PSNCA−/− mice. On 6th day, the pole and grip strength were performed. On 7th day, mice were sacrificed for indicated studies. Following are animal numbers used for these studies: behavioral (n = 10), neurochemical (n = 5), immunohistochemistry (n = 5), and biochemical studies (n = 4) per each treatment group. (PDF 110 kb) [file 13024_2017_233_MOESM2_ESM.pdf]

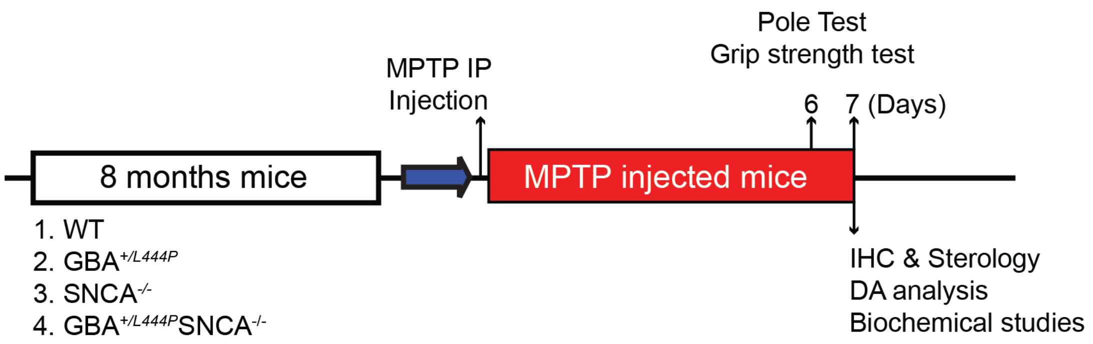

Supplementary Figure 2. The schematic diagram depicts the time schedule of intervention and analyses performed. Numerals represent the days experiments were conducted. On 1th day we injected saline or MPTP (2 h interval, 4 times, 20 mg/kg free base) in 8 months WT, $GBA^{+/L444P}$, $SNCA^{-/-}$, $GBA^{+/L444P}SNCA^{-/-}$ mice. On 6th day, the pole and grip strength were performed. On 7th day, mice were sacrificed for indicated studies. Following are animal numbers used for these studies: behavioral (n=10), neurochemical (n=5), immunohistochemistry (n=5), and biochemical studies (n=4) per each treatment group.
