# Supplementary material for: α-Synuclein accumulation and GBA deficiency due to L444P GBA mutation contributes to MPTP-induced parkinsonism
Source: Mol Neurodegener. 2018 Jan 8;13:1. doi: 10.1186/s13024-017-0233-5 (PMC5759291; doi:10.1186/s13024-017-0233-5)
Supplement: Supplementary file 3 — Effect of α-synuclein deficiency and GBA overexpression on susceptibility of GBA+/L444P mice to MPTP-induced gliosis. a, c Representative images of immunohistochemistry data for GFAP with low (scale bar, 500 μm) and high magnification (scale bar, 50 μm). b, d Intensities of GFAP positive signals in the SNpc of mice treated with saline or MPTP were quantified and shown as a graph. Error bars represent the mean ± S.E.M (n = four mice per group). Two-way ANOVA was used to test for statistical analysis followed by post-hoc Bonferroni test for multiple group comparison. *P < 0.05, ***P < 0.001 vs. MPTP-treated WT or GBA+/L444P with AAV5-Con or MPTP-treated GBA+/L444P with AAV5- Con. n.s: not significant. (PDF 3329 kb) [file 13024_2017_233_MOESM3_ESM.pdf]

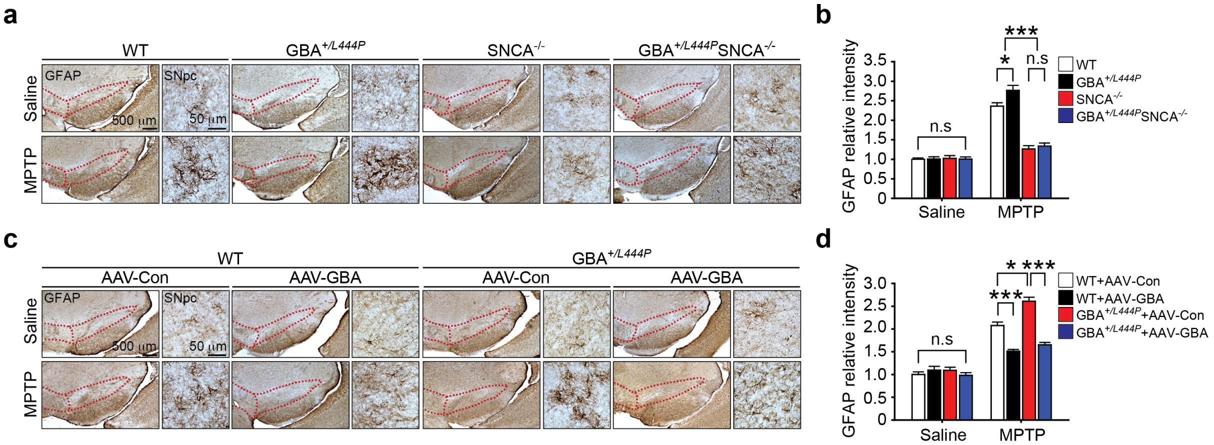

Supplementary Figure 3. Effect of α-synuclein deficiency and GBA overexpression on susceptibility of GBA$^{+/L444P}$ mice to MPTP-induced gliosis. **a**, **c** Representative images of immunohistochemistry data for GFAP with low (scale bar, 500 μm) and high magnification (scale bar, 50 μm). **b**, **d** Intensities of GFAP positive signals in the SNpc of mice treated with saline or MPTP were quantified and shown as a graph. Error bars represent the mean ± S.E.M (n = four mice per group). Two-way ANOVA was used to test for statistical analysis followed by *post-hoc* Bonferroni test for multiple group comparison. *$P < 0.05$, ***$P < 0.001$ vs. MPTP-treated WT or GBA$^{+/L444P}$ with AAV5-Con or MPTP-treated GBA$^{+/L444P}$ with AAV5-Con. n.s: not significant.
