# Supplementary material for: α-Synuclein accumulation and GBA deficiency due to L444P GBA mutation contributes to MPTP-induced parkinsonism
Source: Mol Neurodegener. 2018 Jan 8;13:1. doi: 10.1186/s13024-017-0233-5 (PMC5759291; doi:10.1186/s13024-017-0233-5)
Supplement: Supplementary file 4 — MPTP-induced α-synuclein oligomer. a Filter trap soluble-α- synuclein oligomer species assay from ventral midbrain of WT and GBA+/L444P mice with or without MPTP. Error bars represent the mean ± S.E.M. (n = four mice per group). Two-way ANOVA was used for statistical analysis followed by post-hoc Bonferroni test for multiple group comparison. ***P < 0.001 vs. MPTP-treated WT. N.D: not detection. b Filter trap insoluble-α-synuclein filament species assay from ventral midbrain of WT and GBA+/L444P mice with or without MPTP. α-Synuclein preformed fibril (PFF) is positive control. (PDF 574 kb) [file 13024_2017_233_MOESM4_ESM.pdf]

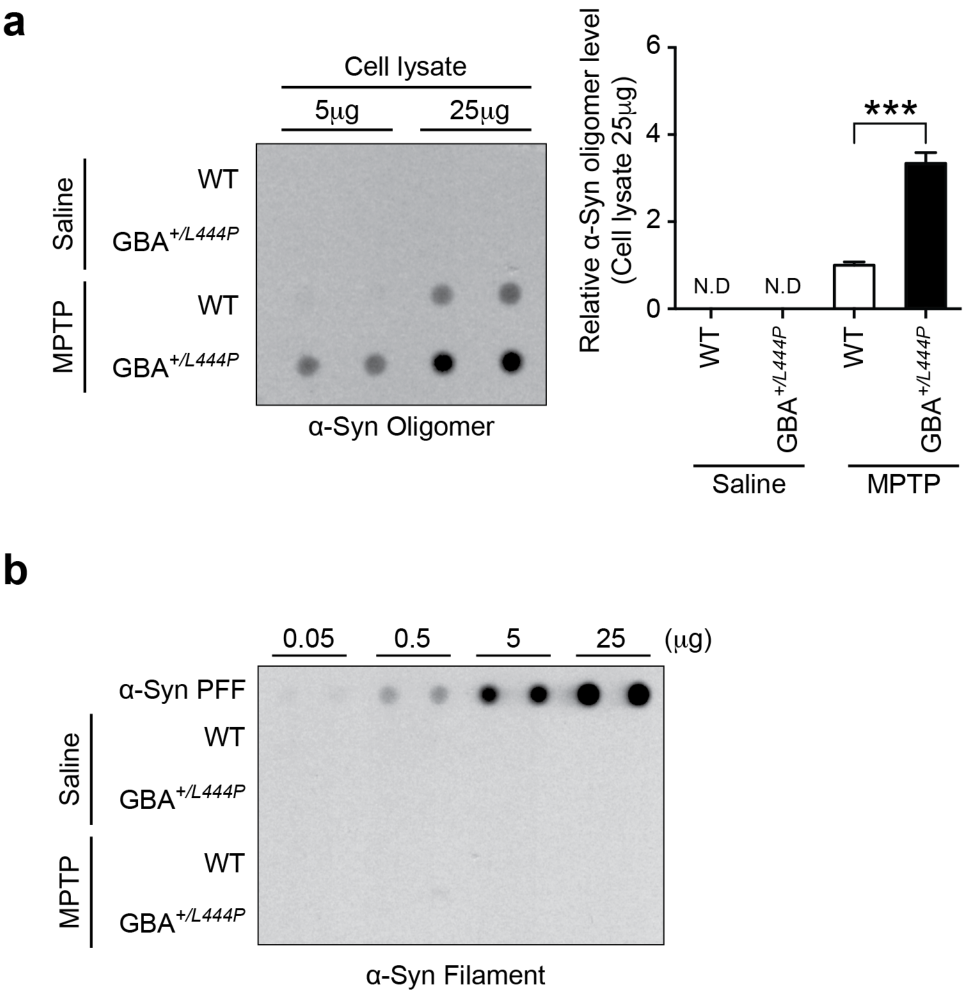

Supplementary Figure 4. MPTP-induced α-synuclein oligomer. **a** Filter trap soluble-α-synuclein oligomer species assay from ventral midbrain of WT and GBA$^{+/L444P}$ mice with or without MPTP. Error bars represent the mean ± S.E.M. (n = four mice per group). Two-way ANOVA was used for statistical analysis followed by *post-hoc* Bonferroni test for multiple group comparison. ***$P < 0.001$ vs. MPTP-treated WT. N.D: not detection. **b** Filter trap insoluble-α-synuclein filament species assay from ventral midbrain of WT and GBA$^{+/L444P}$ mice with or without MPTP. α-Synuclein preformed fibril (PFF) is positive control.
