# Supplementary material for: α-Synuclein accumulation and GBA deficiency due to L444P GBA mutation contributes to MPTP-induced parkinsonism
Source: Mol Neurodegener. 2018 Jan 8;13:1. doi: 10.1186/s13024-017-0233-5 (PMC5759291; doi:10.1186/s13024-017-0233-5)
Supplement: Supplementary file 5 — Lysosomal calcium concentration. SH-SY5Y cells were transfected with indicated constructs for 48 h. The cells were then labeled with CellLight® Lysosome-RFP (LAMP1; red) and loaded with 0.1 mg/ml of lysosomal calcium indicator Oregon Green BAPTA-1 dextran (BAPTA-1; green) for 12 h. The Oregon Green BAPTA-1 signals that co-localized to lysosome (LAMP-1; red) were used for measuring lysosomal calcium concentration. [Ca2+]lys was measured using ratiometric methods via confocal microscopy. Two-way ANOVA was used to test for statistical analysis followed by post-hoc Bonferroni test for multiple group comparison. ***P < 0.001. (PDF 1122 kb) [file 13024_2017_233_MOESM5_ESM.pdf]

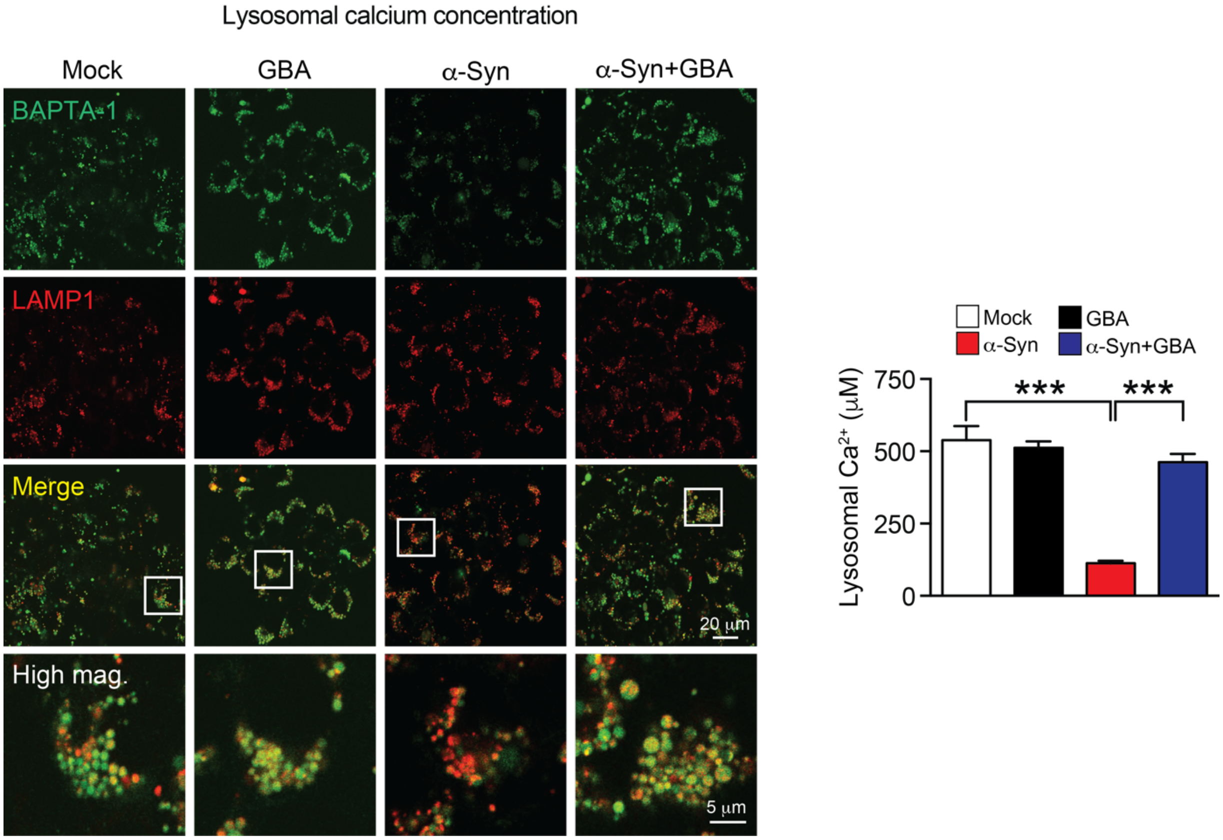

Supplementary Figure 5. Lysosomal calcium concentration. SH-SY5Y cells were transfected with indicated constructs for 48 h. The cells were then labeled with CellLight® Lysosome-RFP (LAMP1; red) and loaded with 0.1 mg/ml of lysosomal calcium indicator Oregon Green BAPTA-1 dextran (BAPTA-1) for 12 h. $[Ca^{2+}]_{lys}$ was measured using ratiometric methods via confocal microscopy. Two-way ANOVA was used to test for statistical analysis followed by *post-hoc* Bonferroni test for multiple group comparison. ***$P < 0.001$.
