# Supplementary material for: α-Synuclein accumulation and GBA deficiency due to L444P GBA mutation contributes to MPTP-induced parkinsonism
Source: Mol Neurodegener. 2018 Jan 8;13:1. doi: 10.1186/s13024-017-0233-5 (PMC5759291; doi:10.1186/s13024-017-0233-5)
Supplement: Supplementary file 6 — The schematic diagram depicts the time AAV5 injection schedule of intervention and analyses performed. Numerals represent the days’ experiments were conducted. For stereotaxic injection of AAV5-GFP and AAV5-hGBA, 8-month-old mice of indicated genotypes were anesthetized with pentobarbital (60 mg/kg). An injection cannula (26.5 gauge) was stereotaxically applied to the substantia nigra pars compacta (SNpc). After AAV5 stereotaxic injection for 1 month, we injected saline or MPTP (2 h interval, 4 times, 20 mg/kg free base) in WT, and GBA+/L444P mice. On 6th day the pole and grip strength test were performed. On 7th day, mice were sacrificed for indicated studies. Following are animal numbers used for these studies: behavioral (n = 6-8), neurochemical (n = 4), immunohistochemistry (n = 4), and biochemical studies (n = 4) per each treatment group. (PDF 126 kb) [file 13024_2017_233_MOESM6_ESM.pdf]

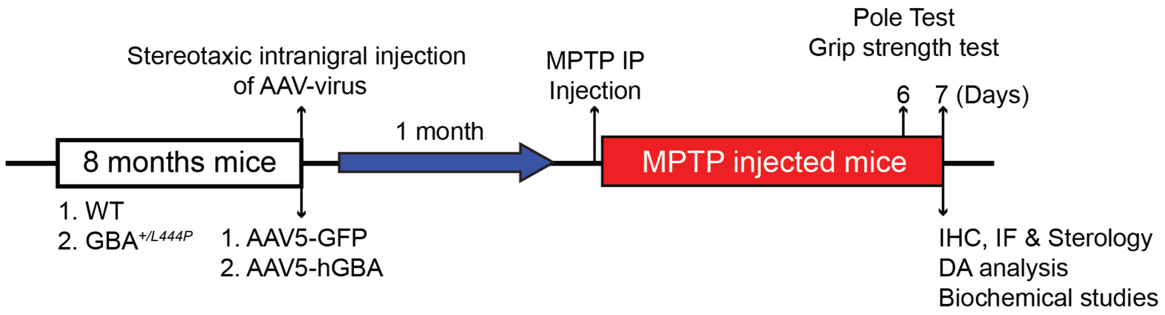

Supplementary Figure 6. The schematic diagram depicts the time AAV5 injection schedule of intervention and analyses performed. Numerals represent the days' experiments were conducted. For stereotaxic injection of AAV5-GFP and AAV5-hGBA, 8-month-old mice of indicated genotypes were anesthetized with pentobarbital (60 mg/kg). An injection cannula (26.5 gauge) was stereotaxically applied to the substantia nigra pars compacta (SNpc). After AAV5 stereotaxic injection for 1 month, we injected saline or MPTP (2 h interval, 4 times, 20 mg/kg free base) in WT, and GBA$^{+/L444P}$ mice. On 6th day the pole and grip strength test were performed. On 7th day, mice were sacrificed for indicated studies. Following are animal numbers used for these studies: behavioral (n=6-8), neurochemical (n=4), immunohistochemistry (n=4), and biochemical studies (n=4) per each treatment group.
