# Supplementary material for: α-Synuclein accumulation and GBA deficiency due to L444P GBA mutation contributes to MPTP-induced parkinsonism
Source: Mol Neurodegener. 2018 Jan 8;13:1. doi: 10.1186/s13024-017-0233-5 (PMC5759291; doi:10.1186/s13024-017-0233-5)
Supplement: Supplementary file 7 — Neuron specific AAV5-hGBA overexpression in SNpc region. a Vector design of AAV5 hGBA. b Representative immunofluorescent images of GFP (green, injection marker), GFAP (red, astrocyte, non-neuronal marker), Tuj1 (Violet, neuronal marker), and DAPI (Blue). (PDF 4671 kb) [file 13024_2017_233_MOESM7_ESM.pdf]

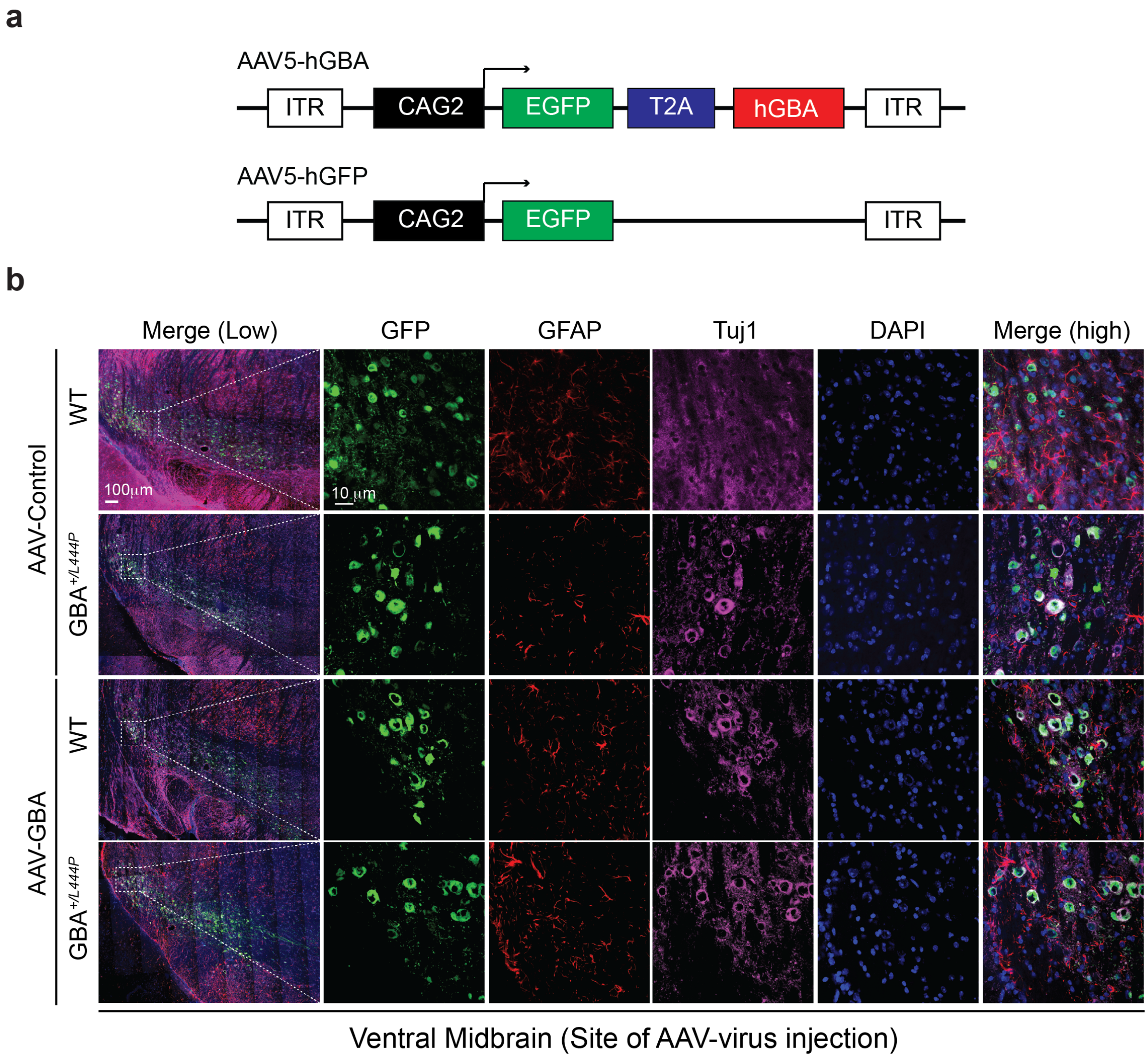

Supplementary Figure 7. Neuron specific AAV5-hGBA overexpression in SNpc region. **a** Vector design of AAV5 hGBA. **b** Representative immunofluorescent images of GFP (green, injection marker), GFAP (red, astrocyte, non-neuronal marker), Tuj1 (Violet, neuronal marker), and DAPI (Blue).
