# Supplementary material for: α-Synuclein accumulation and GBA deficiency due to L444P GBA mutation contributes to MPTP-induced parkinsonism
Source: Mol Neurodegener. 2018 Jan 8;13:1. doi: 10.1186/s13024-017-0233-5 (PMC5759291; doi:10.1186/s13024-017-0233-5)
Supplement: Supplementary file 8 — GBA overexpression inhibits MPTP-reduced mitochondrial protein level in GBA+/L444P mice. a Immunoblots of SDHA, PDH, VDAC, TH, GBA, and α-synuclein from AAV5-Con injected WT, AAV5-hGBA injected WT, AAV5-Con injected heterozygous, and AAV5-hGBA injected heterozygous mice treated with saline or MPTP. VMB lysates were immunoblotted with anti-SDHA, anti-PDH, anti-VDAC, anti-TH, and anti-GBA antibodies. b SDHA, c PDH, d VDAC, e TH, f GBA, and g α-synuclein expression levels were normalized against β-actin. Error bars represent the mean ± S.E.M. (n = three mice per group). Two-way ANOVA was used for statistical analysis followed by post-hoc Bonferroni test for multiple group comparison. *P < 0.05, **P < 0.01, ***P < 0.001 vs. saline-treated WT with AAV-Con or saline-treated GBA+/L444P with AAV5-Con or MPTP-treated WT with AAV5-Con or MPTP-treated GBA+/L444P with AAV5-Con group. (PDF 683 kb) [file 13024_2017_233_MOESM8_ESM.pdf]

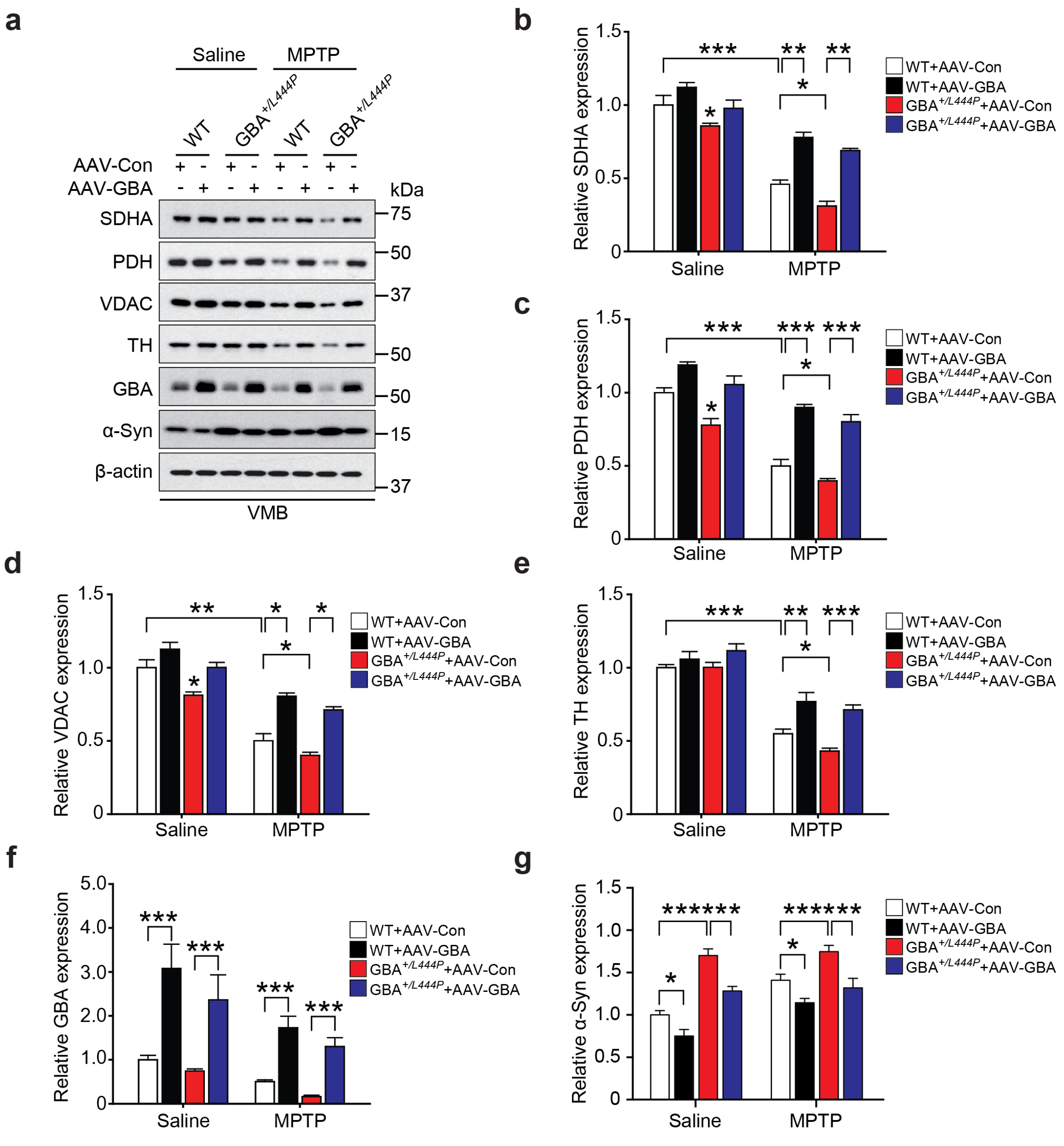

Supplementary Figure 8. GBA overexpression inhibits MPTP-reduced mitochondrial protein level in GBA$^{+/L444P}$ mice. **a** Immunoblots of SDHA, PDH, VDAC, TH, GBA, and α-synuclein from AAV5-Con injected WT, AAV5-hGBA injected WT, AAV5-Con injected heterozygous,

and AAV5-hGBA injected heterozygous mice treated with saline or MPTP. VMB lysates were immunoblotted with anti-SDHA, anti-PDH, anti-VDAC, anti-TH, and anti-GBA antibodies. **b** SDHA, **c** PDH, **d** VDAC, **e** TH, **f** GBA, and **g** α-synuclein expression levels were normalized against β-actin. Error bars represent the mean ± S.E.M. (n = three mice per group). Two-way ANOVA was used for statistical analysis followed by *post-hoc* Bonferroni test for multiple group comparison. *$P < 0.05$, **$P < 0.01$, ***$P < 0.001$ vs. saline-treated WT with AAV-Con or saline-treated GBA$^{+/L444P}$ with AAV5-Con or MPTP-treated WT with AAV5-Con or MPTP-treated GBA$^{+/L444P}$ with AAV5-Con group.
