# Supplementary material for: α-Synuclein accumulation and GBA deficiency due to L444P GBA mutation contributes to MPTP-induced parkinsonism
Source: Mol Neurodegener. 2018 Jan 8;13:1. doi: 10.1186/s13024-017-0233-5 (PMC5759291; doi:10.1186/s13024-017-0233-5)
Supplement: Supplementary file 9 — L444P GBA heterozygous mutation leads to autophagy abnormality. a, b Effect of rapamycin on GBA+/L444P expression. Primary cortical neurons were cultured from WT and GBA+/L444P mice. After 10 DIV, primary neurons were treated with 20 nM of rapamycin (mTOR inhibitor for inducing autophagy) for 24 h. a Representative Immunoblots of GBA. b GBA expression levels were normalized against β-actin and the error bars represent the mean ± S.E.M (n = four per group). c Immunoblots of Autophagy marker proteins SQSTM1/p62, and LC3A/B-I/II. VMB lysates were immunoblotted with anti-SQSTM1/p62, and anti-LC3A/B-I/II antibodies. d SQSTM1/p62, and e LC3A/B-II expression levels were normalized against β-actin. Error bars represent the mean ± S.E.M (n = four mice per group). Two-way ANOVA was used for statistical analysis followed by post-hoc Bonferroni test for multiple group comparison. *P < 0.05, **P < 0.01, ***P < 0.001. n.s: not significant. (PDF 338 kb) [file 13024_2017_233_MOESM9_ESM.pdf]

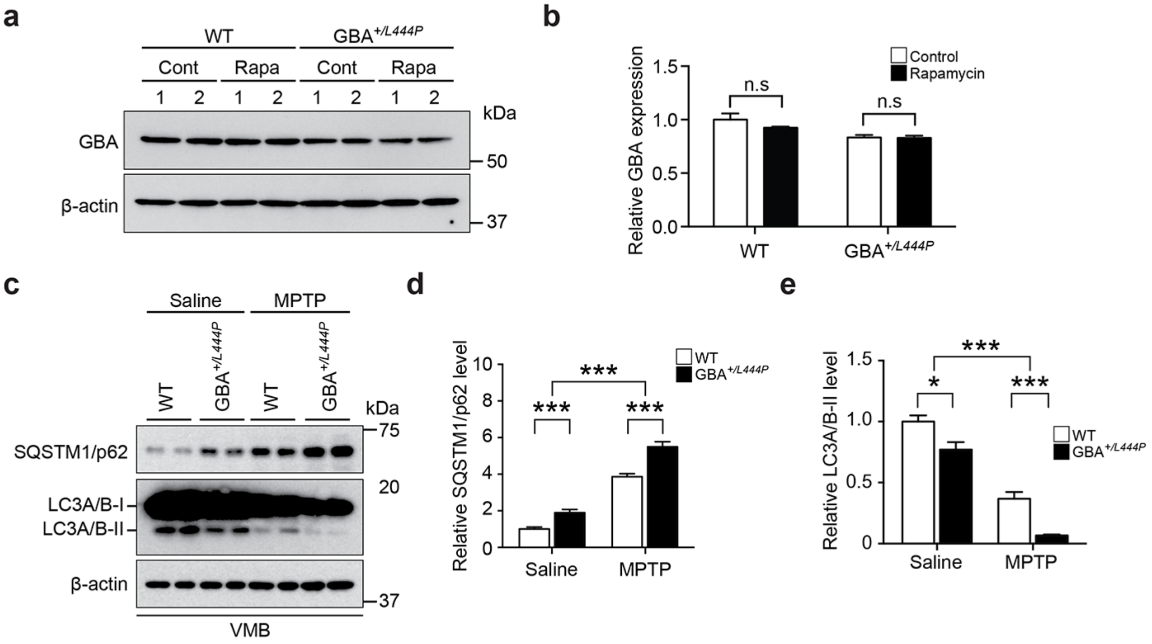

Supplementary Figure 9. L444P GBA heterozygous mutation leads to autophagy abnormality. **a**, **b** Effect of rapamycin on GBA$^{+/L444P}$ expression. Primary cortical neurons were cultured from WT and GBA$^{+/L444P}$ mice. After 10 DIV, primary neurons were treated with 20 nM of rapamycin (mTOR inhibitor for inducing autophagy) for 24 h. **a** Representative Immunoblots of GBA. **b** GBA expression levels were normalized against β-actin and the error bars represent the mean ± S.E.M (n = four per group). **c** Immunoblots of Autophagy marker proteins SQSTM1/p62, and LC3A/B-I/II. VMB lysates were immunoblotted with anti-SQSTM1/p62, and anti-LC3A/B-I/II antibodies. **d** SQSTM1/p62, and **e** LC3A/B-II expression levels were normalized against β-actin. Error bars represent the mean ± S.E.M (n = four mice per group). Two-way ANOVA was used for statistical analysis followed by *post-hoc* Bonferroni test for multiple group comparison. *$P < 0.05$, **$P < 0.01$, ***$P < 0.001$. n.s: not significant.
